# IGFBP5 Restores Endometrial Receptivity and Rescues Implantation Failure in Polycystic Ovary Syndrome

**DOI:** 10.1002/advs.202520455

**Published:** 2026-03-12

**Authors:** Baoying Liao, Chuyu Yun, Hongying Shan, Yuqian Wang, Xiunan Chen, Weisi Lian, Tianliu Peng, Min Zhao, Xunsi Qin, Kailun Hu, Ping Zhou, Yue Wang, Yanli Pang, Rong Li

**Affiliations:** ^1^ State Key Laboratory of Female Fertility Promotion, Center for Reproductive Medicine, Department of Obstetrics and Gynecology, Peking University Third Hospital National Clinical Research Center for Obstetrics and Gynecology, Peking University Third Hospital Key Laboratory of Assisted Reproduction, Peking University, Ministry of Education Beijing Key Laboratory of Collaborative Innovation in Frontier Technologies for Population Quality Beijing China; ^2^ Reproductive Medicine Department The First Affiliated Hospital of Shihezi University Shihezi China; ^3^ Center for Precision Medicine Multi‐Omics Research Institute of Advanced Clinical Medicine Peking University Beijing China; ^4^ Beijing Advanced Center of Cellular Homeostasis and Aging‐Related Diseases Institute of Advanced Clinical Medicine Peking University Beijing China

**Keywords:** endometrial organoids, endometrial receptivity, IGFBP5, IL‐22‐STAT3 signaling pathway, polycystic ovary syndrome

## Abstract

Polycystic ovary syndrome (PCOS) is a metabolic disorder and a major cause of infertility, affecting approximately 5–18% of women of reproductive age worldwide. Emerging evidence suggests that intrinsic endometrial defects play a critical role in PCOS‐associated infertility; however, the underlying molecular mechanisms remain poorly understood. In this study, we demonstrated that interleukin 22 (IL‐22) signaling directly modulated endometrial function and that downregulation of the IL‐22‐STAT3 pathway contributed to impaired endometrial receptivity in PCOS. Consistently, exogenous IL‐22 administration in a PCOS‐like mouse model restored STAT3 phosphorylation and effectively alleviated implantation failure by enhancing endometrial receptivity. Mechanistically, we identified insulin‐like growth factor‐binding protein 5 (IGFBP5) as a direct downstream target of STAT3 in endometrial organoids derived from patients with PCOS. Knockdown of IGFBP5 in the uteri of PCOS‐like mice abolished the beneficial effects of IL‐22, whereas IGFBP5 supplementation restored endometrial receptivity and rescued implantation. Collectively, we showed that the suppression of the IL‐22‐STAT3‐IGFBP5 axis is a key contributor to impaired endometrial receptivity in PCOS, providing a potential therapeutic target for improving pregnancy outcomes via IGFBP5 supplementation.

## Graphical abstract

Impaired endometrial receptivity contributes to the poor pregnancy outcomes in women with PCOS. The authors found that decreased endometrial IL‐22 levels and disrupted STAT3‐IGFBP5 signaling pathway contributed to impaired endometrial receptivity. Supplementation with IL‐22 or IGFBP5 exerts a protective effect on implantation failure in PCOS‐like mice, which provides promising therapeutic targets for PCOS patients. Created in BioRender. Liao, B. (2026) https://BioRender.com/u01hjv5.

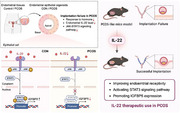

**Abbreviations**
PCOS: Polycystic ovary syndrome
CON: Control
STAT3: Signal transducer and activator of transcription 3
IL‐22: Interleukin 22
IGFBP5: Insulin‐like growth factor binding protein 5
DHEA: Dehydroepiandrosterone
DHT: Dihydrotestosterone
CK7: Cytokeratin‐7
E‐cad: E‐cadherin
DEGs: Differential expressed genes
KEGG analysis: Kyoto Encyclopedia of Genes and Genomes analysis
GSEA: Gene set enrichment analysis
ERα: Estrogen receptor α
PR: Progesterone receptor
PAEP: Progestagen associated endometrial protein
SPP1: Secreted phosphoprotein 1
IHH: Indian hedgehog signaling molecule
LIF: Leukemia inhibitory factor
HSD17B2: Hydroxysteroid 17‐beta dehydrogenase 2
MSX1: Msh homeobox 1
MSX2: Msh homeobox 2
HAND2: Heart and neural crest derivatives expressed 2
IGFBP1: Insulin‐like growth factor binding protein 1
PRL: Prolactin
MUC1: Mucin 1
AREG: Amphiregulin
HOXA10: Homeobox A10
ISs: Implantation sites
IGFs: Insulin‐like growth factors
IGF1R: Insulin‐like growth factor 1 receptor
CREB: cAMP response element binding protein

## 1 Introduction

Polycystic ovary syndrome (PCOS) is the most prevalent endocrine‐related reproductive disorder among women of reproductive age, affecting approximately 5–18% of this population worldwide [1]. PCOS is primarily characterized by hyperandrogenism, oligo‐ovulation or anovulation, polycystic ovarian morphology, and metabolic dysfunction. Infertility and pregnancy complications remain a leading concern associated with this disorder [2, 3]. In recent years, accumulating evidence has suggested that endometrial dysfunction independently contributes to infertility in patients with PCOS, beyond the well‐established ovulatory abnormalities [4, 5]. Impaired endometrial function has been shown to significantly compromise in vitro fertilization (IVF) outcomes in women with PCOS [6, 7]. However, clinical interventions to improve endometrial function during the window of implantation (WOI) remain limited, as the underlying etiology of this condition remains unclear.

Endometrial receptivity refers to the ability of the endometrium to accept embryo implantation and is determined by estrogen‐ and progesterone‐regulated proliferation and differentiation of endometrial cells [8]. During implantation, maternal signaling, including estrogen receptor (ERα) and progesterone receptor (PR), together with embryonic signals, regulates epithelial cell function and coordinates the implantation process [9]. The initial contact and interaction between luminal epithelial cells and the embryo are essential for successful blastocyst attachment and invasion [10]. Recent single‐cell spatial transcriptomic analyses revealed that the endometrium in PCOS exhibits epithelial hyperplasia and dysfunctional subsets [11], directly linking epithelial defects to impaired endometrial receptivity. However, mechanistic studies on how epithelial dysfunction leads to implantation failure remain limited, hindering the development of targeted interventions for endometrial dysfunction in women with PCOS.

Among factors influencing endometrial function, immunoregulatory cytokines are increasingly recognized as key modulators of implantation [12, 13]. Despite downregulation of chemokines that promote embryo implantation, the pro‐inflammatory state of the PCOS endometrium, characterized by elevated interleukin (IL)‐6, IL‐8, IL‐18, and C reaction protein levels, further impairs endometrial function and receptivity [14, 15], suggesting that immune dysregulation contributes to implantation failure in PCOS. IL‐22, a member of the IL‐10 family, regulates mucosal immunity and tissue homeostasis and has been implicated in pregnancy maintenance [16, 17]. Our previous studies demonstrated that IL‐22 improves insulin resistance and suppresses granulosa cell inflammation, alleviating metabolic and ovarian dysfunction in PCOS [18]. However, whether IL‐22 directly regulates endometrial function and endometrial receptivity in PCOS remains unclear.

In this study, we observed reduced IL‐22 levels in the secretory endometrium of patients with PCOS compared with controls, accompanied by downregulation of the JAK‐STAT3 signaling pathway. To further investigate this, we established endometrial organoids derived from patients with PCOS and a dehydroepiandrosterone (DHEA)‐induced PCOS‐like mouse model. In both models, exogenous IL‐22 supplementation improved endometrial receptivity and promoted embryo implantation by enhancing STAT3 phosphorylation. Endometrial organoid assays demonstrated that STAT3 directly binds to the promoter of insulin‐like growth factor‐binding protein 5 (*IGFBP5*) and positively regulates its transcription. Moreover, IGFBP5 supplementation improved endometrial receptivity in PCOS‐like mice model, highlighting the therapeutic potential of targeting the IL‐22‐STAT3‐IGFBP5 axis to improve fertility in patients with PCOS.

## 2 Results

### 2.1 Women with PCOS Show Abnormal Endometrial Receptivity and IL‐22‐STAT3 Signaling

**FIGURE 1:**
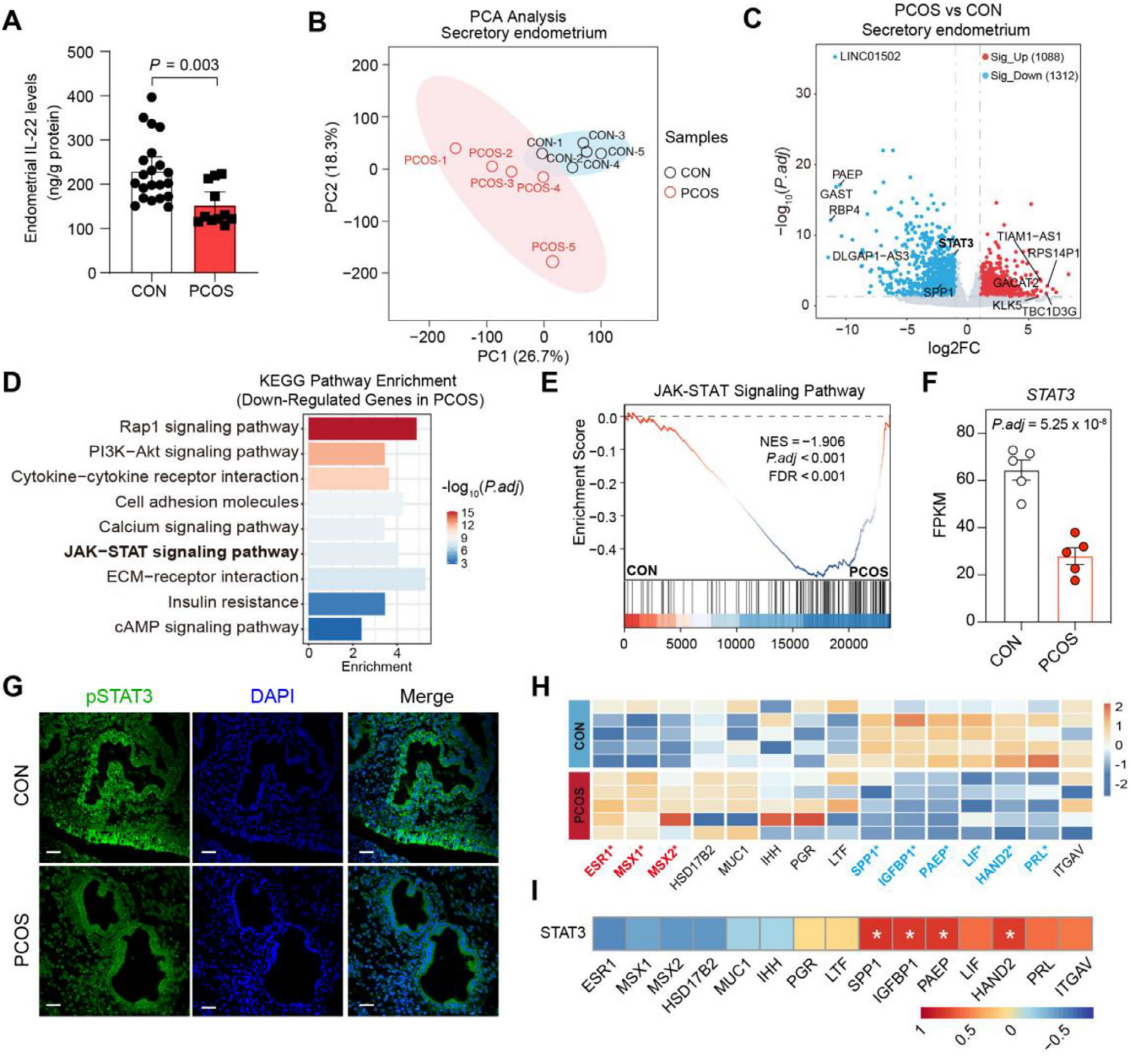
PCOS endometrium is marked by impaired endometrial receptivity and disrupted IL‐22‐STAT3 signaling. (A) IL‐22 levels in secretory endometrium from the CON (*n* = 21) and PCOS (*n* = 11) groups; (B‐C) A PCA plot (B) and a volcano plot (C) for RNA‐seq results in secretory endometrium from the CON and PCOS groups (*n* = 5 per group, |log2FC| > 1, adj. *P* < 0.05 for DEGs); (D) Pathways significantly enriched by KEGG analysis of downregulated DEGs in PCOS secretory endometrium compared with the CON group; (E) Gene set enrichment analysis (GSEA) presents enrichment for JAK‐STAT signaling pathway in PCOS secretory endometrium compared to CON secretory endometrium, NES, normal enrichment score; FDR, false discovery rate; FDR value < 0.05 was considered significant. Significance was calculated by permutation test; (F) RNA‐seq based gene expression values (FPKM) for *STAT3* in control and PCOS secretory endometrium; (G) Representative immunofluorescence staining images of pSTAT3 in secretory endometrium from the CON and PCOS groups, scale bars: 50 µm; (H) Clustered heatmap of endometrial receptivity markers (red for significantly up‐regulated genes *ESR1*, *MSX1*, *MSX2*, blue for down‐regulated genes *PAEP*, *SPP1*, *LIF*, *HAND2*, *IGFBP1*, *PRL*) in PCOS endometrium compared to the CON group, ^*^ indicates differential expressed genes (|log2FC| > 1, adj. *P* < 0.05) between two groups; (I) Correlation analysis between the expression level of STAT3 and endometrial receptivity markers. For A, data are presented as medians with interquartile ranges; the *P* value was determined by a two‐tailed Mann‐Whitney U‐test. For I, ^*^ *P* < 0.05, significance was calculated by Spearman correlation. For G, the experiments were performed in three replicates.

IL‐22 has been implicated in improving insulin resistance and suppressing granulosa cell inflammation in PCOS [18]. However, whether IL‐22 directly modulates endometrial function in PCOS remains unclear. In the present study, we observed that the secretory endometrium of patients with PCOS exhibited significantly lower IL‐22 levels compared with controls (CON) (Figure 1A), suggesting that local IL‐22 deficiency may contribute to endometrial dysfunction in PCOS. To further characterize these abnormalities, we performed RNA sequencing of secretory endometrial samples from patients with PCOS (*n* = 5) and healthy CON (*n* = 5). The endometrium of the PCOS group showed a distinct gene expression profile compared with CON, including marked downregulation of *STAT3* and other positive regulators of endometrial receptivity (Figure 1B,C; Figure S1A). Differentially expressed genes (DEGs) in the endometrium of patients with PCOS were enriched in IL‐22‐related pathways, including the JAK‐STAT signaling pathway and cytokine‐cytokine receptor interactions (Figure 1D,E; Figure S1B). Notably, STAT3 and its phosphorylated form (pSTAT3), a canonical downstream mediator of IL‐22 signaling, were both markedly reduced in the secretory endometrium of patients with PCOS, confirming disruption of the STAT3 pathway (Figure 1F,G; Figure S1C). Given the critical role of estrogen and progesterone in endometrial receptivity, we assessed the expression of their receptors and downstream genes. *ESR1* and the estrogen‐responsive genes *MSX1* and *MSX2* were abnormally upregulated in PCOS, whereas key endometrial receptivity markers, including *PAEP*, *SPP1*, *LIF*, and *HAND2*, were significantly downregulated in the secretory endometrium of patients with PCOS (Figure 1H). Furthermore, STAT3 expression positively correlated with key endometrial receptivity markers (Figure 1I), indicating a critical role for STAT3 in endometrial receptivity. Collectively, these findings suggested that endometrial dysfunction in PCOS was associated with impaired IL‐22‐STAT3 signaling.

### 2.2 IL‐22 Supplementation Restores Uterine Receptivity in PCOS‐Like Mice

**FIGURE 2:**
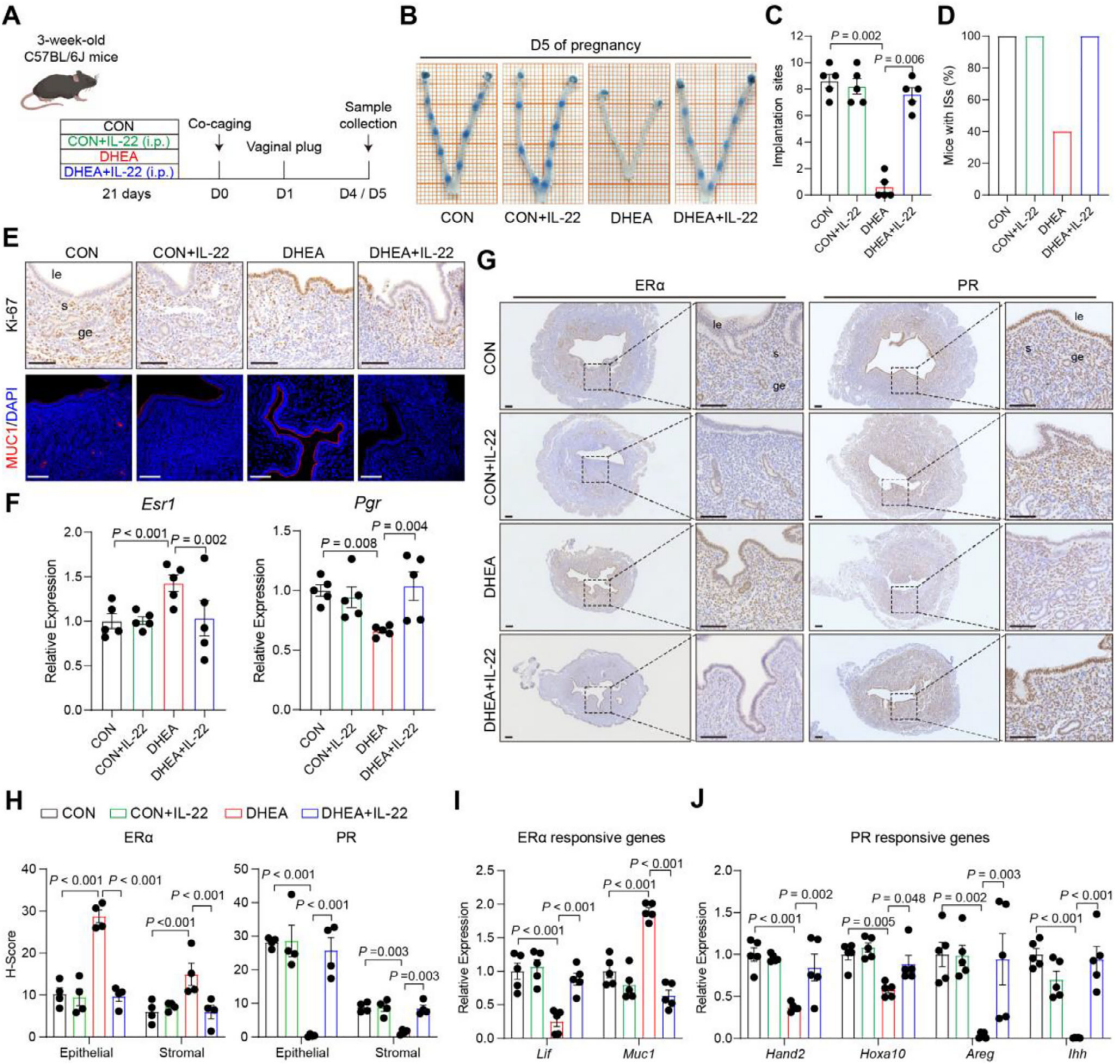
IL‐22 alleviates the implantation disorder of PCOS‐like mice. (A) Experiment design for mice treatment with IL‐22. 3‐week‐old female mice were daily subcutaneously injected with sesame oil or DHEA (6 mg/100 g body weight; dissolved in sesame oil) for 21 consecutive days. For IL‐22 supplementation, PBS or 100 µg/kg of IL‐22 was intraperitoneally injected to control and DHEA mice every day during the three weeks of sesame oil or DHEA treatment. (B) Representative images presenting ISs in CON, CON+IL‐22, DHEA and DHEA + IL‐22 mice; (C) Number of ISs in CON, CON+IL‐22, DHEA and DHEA + IL‐22 mice; (D) Percentage of mice with ISs in mice that had a vaginal plug, *n* = 5 per group; (E) Representative immunohistochemistry staining images of Ki‐67 and immunofluorescence staining images of MUC1 in mice uterus on day 4 of pregnancy, scale bars: 100 µm; (F) Relative gene expression of *Esr1* and *Pgr* in mice uterus on day 4 of pregnancy, *n =* 5 per group; (G‐H) Representative immunohistochemistry staining images (G) and quantitative results (H) of ERα and PR protein in mice uterus on day 4 of pregnancy, *n =* 4 per group, scale bars: 100 µm; (I‐J) Relative gene expression of estrogen responsive genes *Lif* and *Muc1* (I) and progesterone responsive genes *Hand2*, *Hoxa10*, *Areg* and *Ihh* (J) in mice uterus on day 4 of pregnancy, *n =* 5 per group. For C, data is presented as medians with interquartile ranges, the *P* value was determined by Kruskal–Wallis test followed by Dunn's post hoc test. For F, H, I, and J, data are presented as mean ± SEM, the *P* value was determined by one‐way ANOVA with Tukey's multiple comparison post hoc test. For E and G, the experiments were performed in three replicates; le, luminal epithelium; ge, glandular epithelium; s, stroma.

To determine the effect of IL‐22 on uterine receptivity, IL‐22 was administered to CON mice and a DHEA‐induced PCOS‐like mouse model (Figure 2A). No implantation sites (ISs) were found in DHEA‐treated mice on day 5 of pregnancy, whereas IL‐22 administration significantly increased the number of ISs compared with DHEA mice (Figure 2B–D). DHEA‐treated mice exhibited impaired uterine receptivity, characterized by excessive MUC1 expression and increased epithelial proliferation, both of which were partially reversed by IL‐22 treatment (Figure 2E; Figure S2A, B). In CON mice, IL‐22 treatment did not alter the number of ISs or endometrial status compared with untreated CON (Figure 2A–E). We next examined the steroid hormone receptors PR and ERα, key regulators of endometrial receptivity. ERα expression was significantly increased, whereas PR expression was significantly reduced in DHEA‐induced PCOS‐like mice; this aberrant pattern was reversed by IL‐22 supplementation (Figure 2F–H). Likewise, although IL‐22 had little effect in CON mice, it restored the expression of key ERα‐ and PR‐responsive genes, including *Lif*, *Muc1*, *Hand2*, *Hoxa10*, *Areg*, and *Ihh*, in DHEA‐treated mice (Figure 2I,J). These findings suggested that IL‐22 supplementation rescued abnormal uterine receptivity and implantation failure, thereby improving fertility in PCOS‐like mice.

### 2.3 Abnormal Hormonal Responses in PCOS Endometrium‐Derived Epithelial Organoids

**FIGURE 3:**
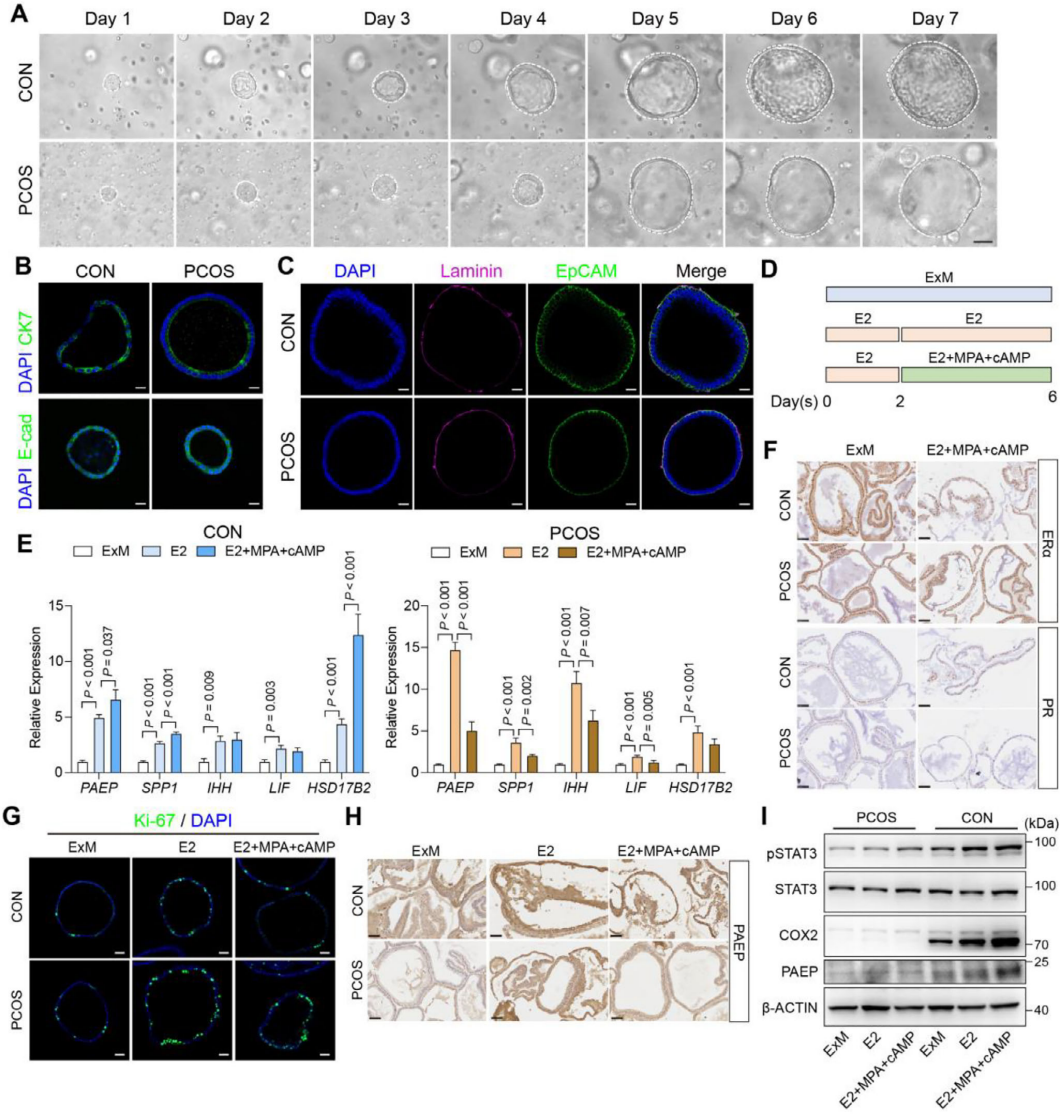
Endometrial organoids simulate the molecular characteristics of the endometrium in vivo. (A) Growth of endometrial epithelial organoids (passage 1) from the CON group and PCOS group, scale bar: 50 µm; (B) Representative immunofluorescence staining pictures of cytokeratin‐7 (CK7) and E‐cadherin (E‐cad) in endometrial organoids, scale bar: 20 µm; (C) Representative immunofluorescence staining of cell basement membrane marker Laminin and EpCAM in endometrial organoids, scale bar: 20 µm; (D) Experiment design for hormone treatment of endometrial organoids: organoids were cultured in expansion medium (ExM) or treated with 10 nM E2 for 6 days, or treated with 10 nM E2 for 2 days and following exposure to 10 nM E2 + 1 μΜ MPA + 1 μΜ cAMP for 4 days; (E) Relative expression of *PAEP*, *SPP1*, *IHH*, *LIF*, and *HSD17B2* in CON and PCOS endometrial organoids during hormone treatment, *n* = 8 per group; (F) Representative immunohistochemistry staining of ERα and PR in the CON and PCOS endometrial organoids, scale bars: 50 µm; (G) Representative immunofluorescence staining pictures of Ki‐67 in the endometrial organoids of the CON and PCOS groups, scale bars: 50 µm; (H) Representative immunohistochemistry staining of PAEP in the CON and PCOS endometrial organoids, scale bars: 50 µm; (I) Representative immunoblots of COX2, PAEP, pSTAT3 and STAT3 in CON and PCOS endometrial organoids under hormone treatment. For E, data are presented as mean ± SEM, the *P* value was determined by one‐way ANOVA with Tukey's multiple comparison post hoc test. For B, C, F, G, and H, the experiments were performed in three replicates.

To investigate the mechanistic role of IL‐22 in human endometrial epithelial cells, we established endometrial organoids from tissues of CON and patients with PCOS [19, 20]. Organoids from both the groups were morphologically comparable and exhibited no differences in epithelial markers (CK7, E‐cadherin) or polarization (Figure 3A–C; Figure S3A–C). The endometrial organoids closely recapitulated the molecular features and secretory functions of their tissues of origin, including glycogen secretion (Figure S3D,E). These results demonstrated that the organoids effectively model the molecular characteristics of the endometrium, providing a powerful tool for studying endometrial epithelial cell function.

During the menstrual cycle, estrogen (E2) and progesterone (P4) regulate cyclic proliferation and differentiation of endometrial cells, which are essential for establishing endometrial receptivity. To assess hormonal responses in organoids, proliferative‐phase‐derived organoids were treated with E2 alone (mimicking the proliferative phase) or E2 followed by E2 + medroxyprogesterone acetate (MPA) + cyclic adenosine monophosphate (cAMP) to stimulate the secretory phase (Figure 3D). PCOS organoids recapitulated key pathological features of the PCOS endometrium, including loss of receptivity markers (*PAEP*, *SPP1*, and *HSD17B2*) after progesterone stimulation, accompanied by ERα upregulation and PR downregulation, suggesting an impaired hormonal response (Figure 3E,F; Figure S3F). We also observed aberrant epithelial proliferation and reduced protein levels of PAEP and COX2 in PCOS organoids (Figure 3G–I; Figure S3G,H). Furthermore, pSTAT3 was significantly reduced (Figure 3I), consistent with the abnormal pSTAT3 levels observed in the secretory endometrium of patients with PCOS. IL‐22 treatment in PCOS organoids promoted STAT3 phosphorylation and *PGR* expression (Figure S3I–K).

Thus, PCOS‐derived epithelial organoids faithfully recapitulated the pathological and functional defects of the endometrium, including aberrant responses to hormones and impaired receptivity, providing a robust platform to study the direct effects of IL‐22. We next used this system to identify downstream mediators of IL‐22‐STAT3 signaling.

### 2.4 IL‐22 Promotes IGFBP5 Expression Through STAT3 Signaling

**FIGURE 4:**
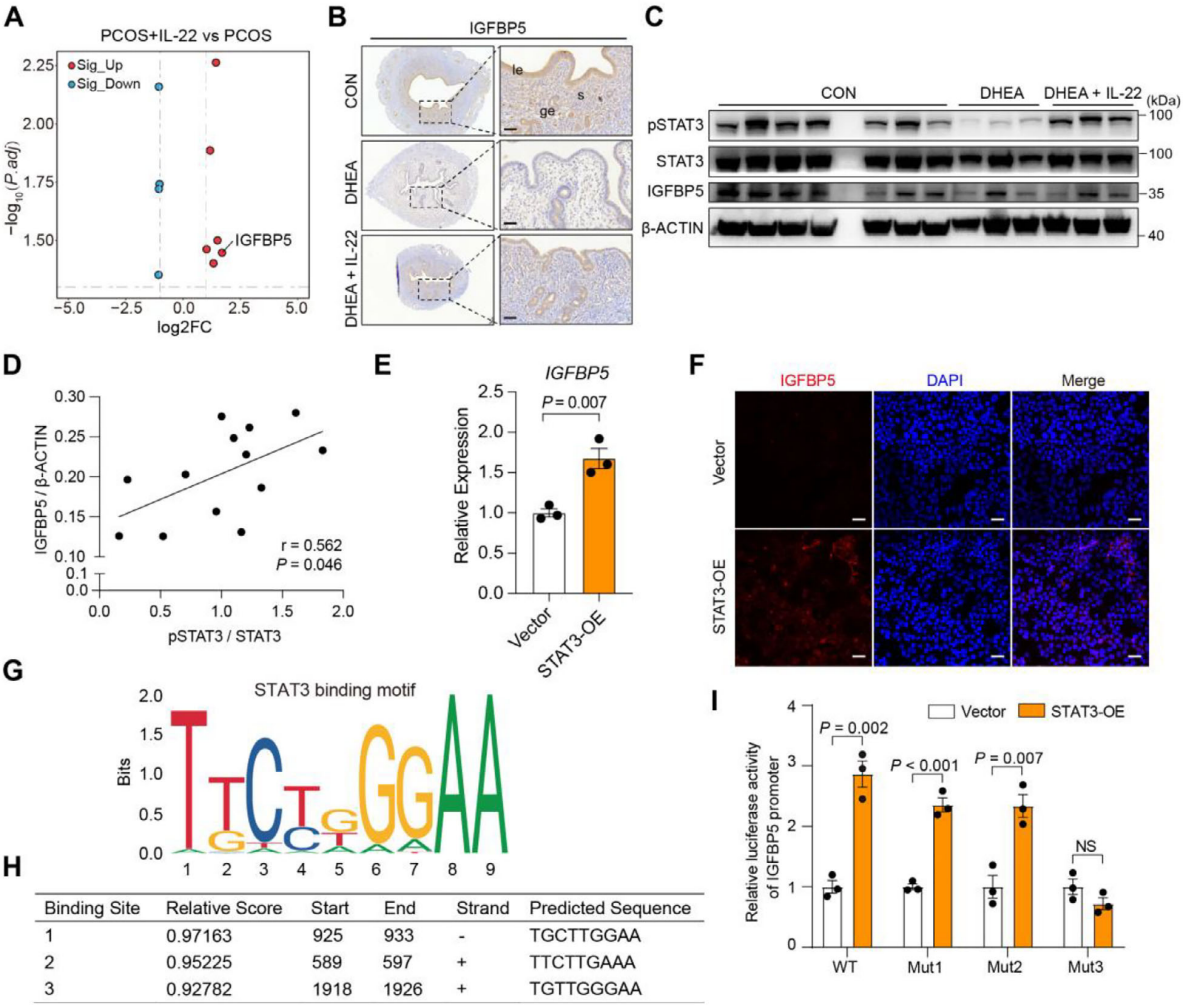
IL‐22 promotes *IGFBP5* expression by activating the STAT3 signaling pathway. (A) Volcano plots showing DEGs in PCOS and PCOS+IL‐22 organoids (|log2FC| > 1, adj. *P* < 0.05), red dot represent for upregulated DEGs, blue dot represent for down‐regulated DEGs, *n =* 3 per group; (B) Immunohistochemistry staining of IGFBP5 in CON, DHEA and DHEA + IL‐22 mice uterus on day 4 of pregnancy; le, luminal epithelium; ge, glandular epithelium; s, stroma, scales: 50 µm; (C) representative immunoblots of CON (*n =* 7), DHEA (*n =* 3) and DHEA + IL‐22 (*n =* 3) mice uterus on day 4 of pregnancy; (D) Spearman correlation analysis between relative IGFBP5 protein levels and pSTAT3/STAT3 levels in mice uterus on day 4 of pregnancy (*n =* 13 in total). (E) qPCR results of *IGFBP5* expression in STAT3‐OE plasmids‐transfected Ishikawa cells, *n =* 3 per group; (F) Representative immunofluorescence staining pictures of IGFBP5 in Ishikawa cells transfected with Vector or STAT3‐OE plasmids, scale bars: 50 µm; (G‐H) Binding motif (G) and predicted specific binding sites (H) of STAT3 and the *IGFBP5* gene promoter; (I) Dual‐luciferase reporter assay of 293T cells, transfected with the indicated plasmids, *n =* 3 per group. For E and I, data are presented as mean ± SEM, the *P* value was determined by a two‐tailed Student's *t*‐test. For B and F, the experiments were performed in three replicates.

Transcriptomic sequencing of PCOS organoids with or without IL‐22 treatment was performed to identify candidate downstream targets of the IL‐22‐STAT3 signaling pathway. Among these, *IGFBP5* was robustly upregulated (Figure 4A). IGFBP5 is a multifunctional protein that regulates the availability of insulin‐like growth factors (IGFs) and is involved in female fertility [21]. In addition, IGFBP5 expression was significantly reduced in PCOS‐like mice (Figure 4B,C; Figure S4A,B). To understand the regulatory relationship between IL‐22 and IGFBP5, IL‐22 was administered intraperitoneally to PCOS‐like mice. IL‐22 supplementation increased both IGFBP5 and pSTAT3 levels in DHEA‐treated mice (Figure 4C). Moreover, IGFBP5 protein levels significantly and positively correlated with pSTAT3 levels (Figure 4D), suggesting that IL‐22 promotes IGFBP5 expression via STAT3 phosphorylation.

To elucidate the mechanistic link between STAT3 signaling and *IGFBP5* expression, we first generated STAT3‐overexpressing (STAT3‐OE) Ishikawa cells, which exhibited significantly increased *IGFBP5* levels (Figure 4E,F; Figure S4C,D), confirming that STAT3 positively regulates *IGFBP5* expression. We then investigated how STAT3 promotes *IGFBP5* expression. Using JASPAR, we identified specific STAT3 binding sites in the *IGFBP5* promoter (Figure 4G,H). Subsequently, we constructed *IGFBP5* promoter reporter plasmids that either contained the wild‐type promoter or point mutations in the three highest‐scoring predicted binding sites (Figure 4H). Luciferase assays demonstrated that mutation of site 3 abolished STAT3‐mediated activation of *IGFBP5* expression (Figure 4I). Collectively, these results demonstrated that STAT3 acted as a key transcription factor downstream of IL‐22 signaling, directly promoting *IGFBP5* expression by binding to its promoter.

### 2.5 IL‐22 Requires IGFBP5 to Improve Endometrial Receptivity in PCOS

**FIGURE 5:**
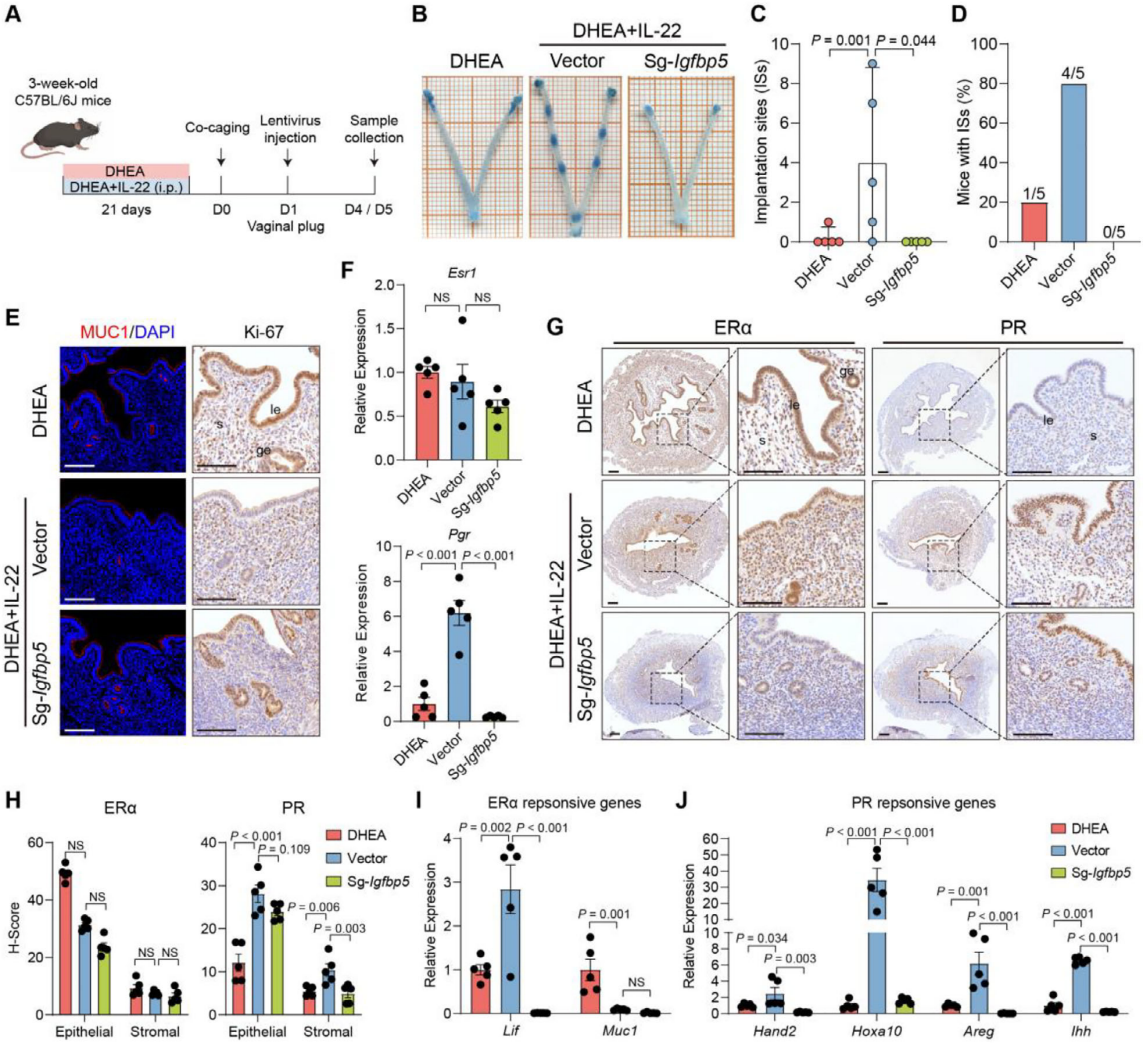
IL‐22 acts through IGFBP5 to alleviate implantation disorders in PCOS. (A) Experiment design for mice treatment and intrauterine injection of lentivirus; (B) Images presenting implantation sites (ISs) in three groups; (C) Number of ISs in three groups, *n =* 5 per group; (D) Percentage of mice with ISs in mice that had a vaginal plug; (E) Representative immunofluorescence staining of MUC1 and immunohistochemistry staining of Ki‐67 in mice uterus on day 4 of pregnancy, *n =* 5 per group, scale bars: 100 µm; (F) Relative gene expression of *Esr1* and *Pgr* in mice uterus on day 4 of pregnancy, *n =* 5 per group; (G‐H) Representative immunohistochemistry staining images (G) and quantitative results (H) of ERα and PR protein in mice uterus on day 4 of pregnancy, *n =* 5 per group, scale bars: 100 µm; (I‐J) Relative gene expression of estrogen responsive genes *Lif* and *Muc1* (I) and progesterone responsive genes *Hand2*, *Hoxa10*, *Areg* and *Ihh* (J) in mice uterus on day 4 of pregnancy, *n =* 5 per group. For C, data is presented as medians with interquartile ranges, and the *P* value was determined by Kruskal–Wallis test followed by Dunn's post hoc test. For F, H‐J, data are presented as mean ± SEM, the *P* value was determined by one‐way ANOVA with Tukey's multiple comparison post hoc test. For E and G, the experiments were performed in three replicates; le, luminal epithelium; ge, glandular epithelium; s, stroma.

To validate that IL‐22‐STAT3 regulates fertility via IGFBP5, we performed intrauterine injection of an *Igfbp5* lentivirus in DHEA+IL‐22‐treated mice on day 1 (Figure 5A), which effectively reduced IGFBP5 levels (Figure S5A–D). After mating with fertile mice, *Igfbp5* knockdown mice showed no ISs on day 5 (Figure 5B–D) and exhibited uterine dysfunction with abnormal epithelial proliferation (Figure 5E; Figure S5E,F), indicating that IL‐22‐mediated improvement of endometrial receptivity depends on IGFBP5. *Igfbp5* knockdown downregulated PR expression but had little impact on ERα levels (Figure 5F–H). Consistently, ERα‐responsive gene *Muc1* remained unaffected, whereas PR target genes were significantly suppressed by *Igfbp5* knockdown (Figure 5I,J), highlighting differential regulation of ERα and PR by IGFBP5 in DHEA+IL‐22‐treated mice. Collectively, these findings revealed a mechanistic dependence of IL‐22 on IGFBP5 to improve endometrial receptivity in PCOS‐like mice.

### 2.6 IGFBP5 contributes to the Establishment of Endometrial Receptivity in Patients with PCOS

**FIGURE 6:**
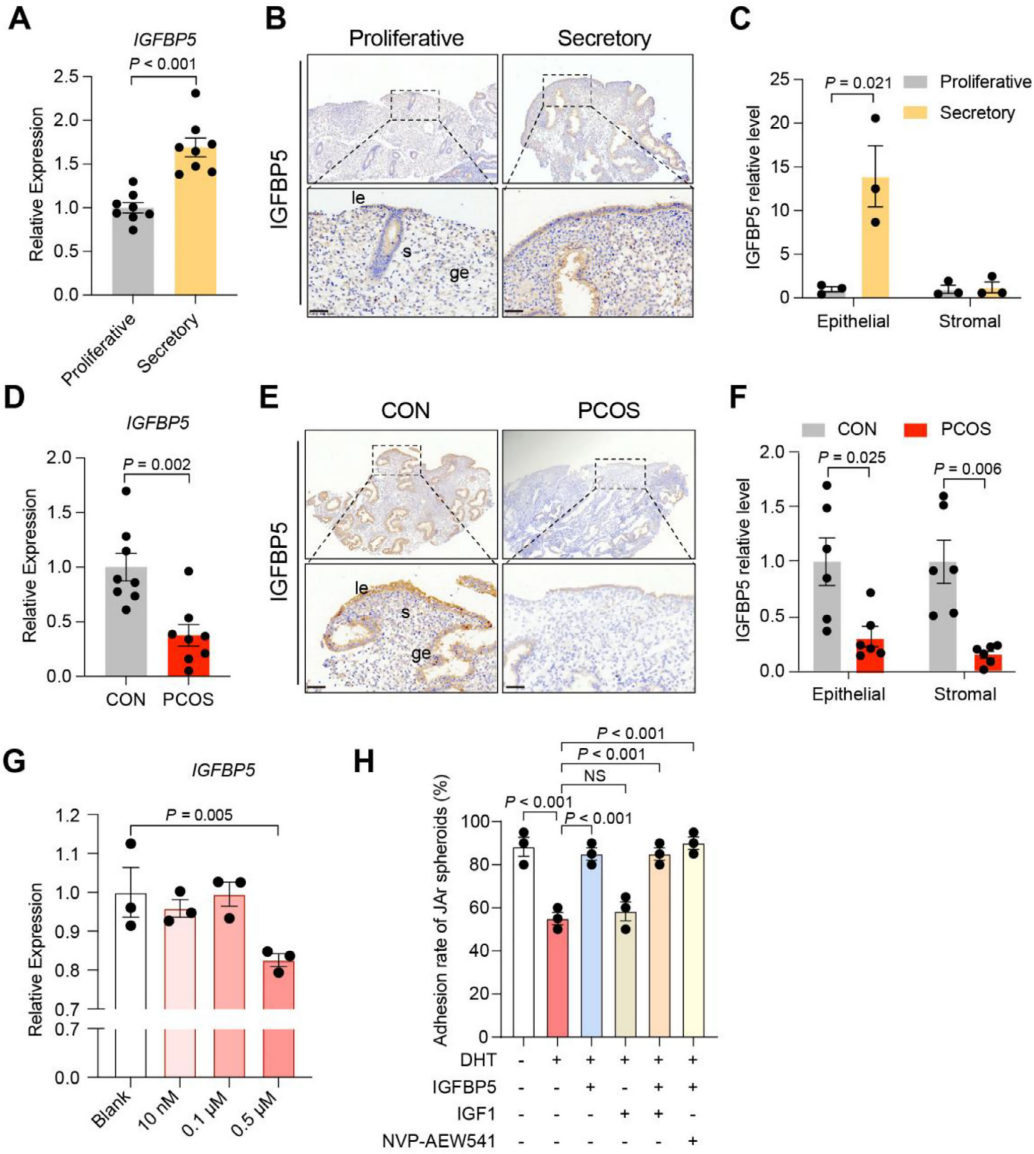
IGFBP5 supplementation improves endometrial receptivity in PCOS‐derived endometrium. (A) Relative expression of *IGFBP5* in proliferative and secretory endometrium of control women, *n =* 8 per group; (B) Representative immunohistochemistry staining of IGFBP5 in human endometrium of proliferative and secretory phase of control women, scale bars: 50 µm; (C) Quantitative analysis of IGFBP5 protein levels in epithelial and stromal cells of proliferative and secretory endometrium of control women, *n =* 3 per group; (D) Relative expression of *IGFBP5* in secretory endometrium from the CON and PCOS groups, *n =* 8 per group; (E‐F) Representative immunohistochemistry staining (E) and its quantitative analysis (F) of IGFBP5 protein levels in the secretory endometrium from the CON and PCOS groups, *n =* 6 per group, scale bars: 50 µm; (G) *IGFBP5* mRNA levels in DHT‐treated Ishikawa cells, *n =* 3 per group; (H) The percentage of JAr spheroids that adhere to Ishikawa cells treated with DHT (0.5 µM), DHT + IGFBP5 (100 ng/ml), DHT + IGF1 (50 ng/ml), DHT + IGFBP5 + IGF1 and DHT + IGFBP5 + NVP‐AEW541 (10 µM), *n =* 3 per group. For A, C, D, F, G, and H, data are presented as mean ± SEM. For A, C, D, and F, the *P* value was determined by a two‐tailed Student's *t*‐test; for G and H, the *P* value was determined by one‐way ANOVA with Tukey's multiple comparison post hoc test. For B and E, the experiments were performed in three replicates; le, luminal epithelium; ge, glandular epithelium; s, stroma.

In endometrium from control women, IGFBP5 levels were significantly lower in the proliferative phase, suggesting that IGFBP5 primarily functions during the secretory phase (Figure 6A–C) and may contribute to the establishment of endometrial receptivity. Notably, IGFBP5 expression was significantly reduced in both epithelial and stromal cells of patients with PCOS (Figure 6D–F). Subsequently, to mimic the androgen‐excess environment of the PCOS endometrium, Ishikawa cells were treated with increasing doses of dihydrotestosterone (DHT), which significantly inhibited *IGFBP5* expression at 0.5 µM DHT (Figure 6G). To further investigate the role of IGFBP5 in implantation, an in vitro model was subsequently applied. JAr spheroids failed to adhere to DHT‐treated Ishikawa cells, whereas exogenous IGFBP5 markedly restored adhesion (Figure 6H). In contrast, IGF1 had little impact on spheroid adhesion, and treatment with IGF1 or the IGF1 receptor (IGF1R) inhibitor NVP‐AEW541 did not alter the IGFBP5‐mediated improvement (Figure 6H), implying that IGFBP5 restores adhesion in an IGF‐independent manner.

These results indicated that epithelial IGFBP5 was downregulated under PCOS and androgen‐excess conditions and played a critical role in maintaining endometrial receptivity in vitro.

### 2.7 IGFBP5 Supplementation Restores Endometrial Receptivity in DHEA‐Induced PCOS‐like Mice

**FIGURE 7:**
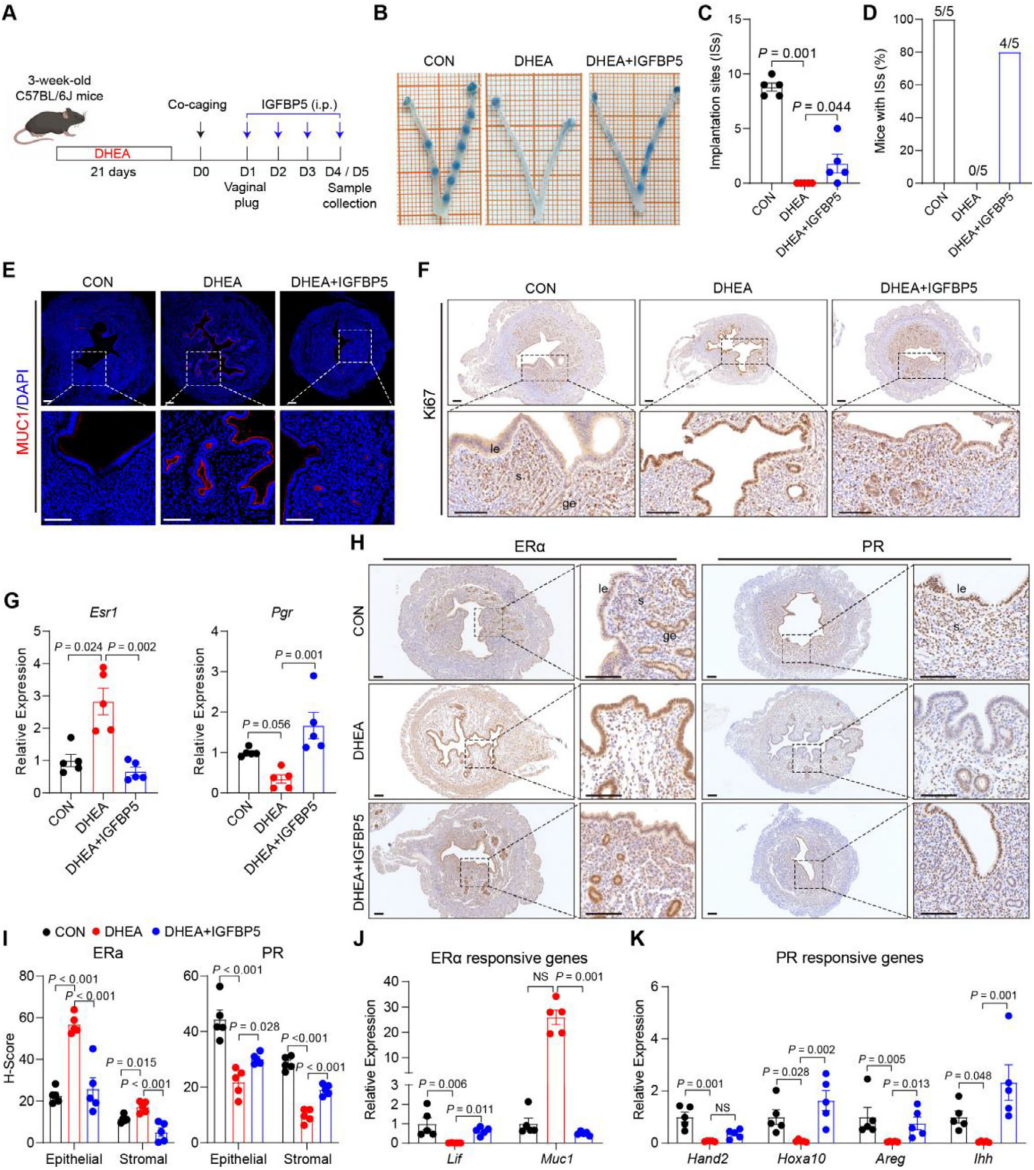
IGFBP5 alleviates the implantation disorder of PCOS‐like mice. (A) Experiment design for mice treatment; (B) Images presenting ISs in CON, DHEA and DHEA +IGFBP5 mice; (C) Number of ISs in CON, DHEA and DHEA + IL‐22 mice, *n =* 5 per group; (D) Percentage of mice with ISs in mice that had a vaginal plug; (E‐F) Representative immunofluorescence staining of MUC1 (E) and immunohistochemistry staining of Ki‐67 (F) in mice uterus on day 4 of pregnancy, scale bars: 100 µm; (G) Relative gene expression of *Esr1* and *Pgr* in mice uterus on day 4 of pregnancy, *n =* 5 per group; (H‐I) Representative immunohistochemistry staining (H) and quantitative results (I) of ERα and PR protein in mice uterus on day 4 of pregnancy, scale bars: 100 µm, *n =* 5 per group; (J‐K) Relative gene expression of estrogen responsive genes *Lif* and *Muc1* (J) and progesterone responsive genes *Hand2*, *Hoxa10*, *Areg* and *Ihh* (K) in mice uterus on day 4 of pregnancy, *n =* 5 per group. For C, data is presented as medians with interquartile ranges, the *P* value was determined by Kruskal–Wallis test followed by Dunn's post hoc test. For G and I‐K, data are presented as mean ± SEM, the *P* value was determined by one‐way ANOVA with Tukey's multiple comparison post hoc test. For E, F, and H, the experiments were performed in three replicates; le, luminal epithelium; ge, glandular epithelium; s, stroma.

To further validate the role of IGFBP5 in endometrial receptivity, DHEA‐induced PCOS‐like mice were intraperitoneally injected with IGFBP5 for four consecutive days starting on day 1 (Figure 7A). IGFBP5 supplementation significantly promoted embryo implantation, as shown by an increased number of ISs and a higher percentage of mice with ISs (Figure 7B–D). Uterine morphology was restored after IGFBP5 supplementation, characterized by reduced MUC1 levels and epithelial cell proliferation (Figure 7E–F; Figure S6A,B). In addition, IGFBP5 rescued the abnormal expression of ERα, PR, and their downstream target genes in DHEA‐treated mice (Figure 7G–K), supporting its role in improving endometrial receptivity.

To explore IGF‐independent mechanisms underlying IGFBP5 function, Ishikawa cells were treated with IGFBP5. IGFBP5 has been reported to signal through cAMP response element‐binding protein (CREB), a key transcription factor in cAMP signaling that plays a vital role in embryo implantation and decidualization [22, 23, 24, 25]. Moreover, cAMP response element (CRE) sites are present in the *PGR* promoter [26]. Our results showed that IGFBP5 supplementation significantly increased phosphorylated CREB levels, enhancing *PGR* expression while having little effect on *ESR1* expression (Figure S6C,D). In addition, CREB phosphorylation was significantly reduced in the uteri of DHEA‐treated mice, and IGFBP5 administration restored CREB activation (Figure S6E), demonstrating the involvement of IGFBP5‐CREB signaling in modulating endometrial function.

Taken together, these results indicated that IGFBP5 enhanced endometrial receptivity and rescued implantation failure in PCOS‐like mice.

## 3 Discussion

Implantation failure due to abnormal endometrial receptivity is a major cause of poor pregnancy outcomes in women with PCOS, yet its etiology remains unclear. IL‐22 has been reported to alleviate insulin resistance and ovarian inflammation in PCOS [18]; however, its direct role in the endometrium had not been explored. To investigate the mechanisms underlying endometrial dysfunction in PCOS, we conducted a comprehensive analysis using clinical samples, PCOS‐like mice, and organoids derived from patients with PCOS, allowing for robust conclusions despite differences in human and mouse endometrial physiology and the limitations of the DHEA‐induced mouse model [27]. We found that IL‐22‐STAT3 signaling was disrupted in the PCOS endometrium. More importantly, we uncovered a previously unrecognized IL‐22‐STAT3‐IGFBP5 axis that directly governs endometrial receptivity, distinguishing our findings from prior studies that primarily focused on metabolic and ovarian mechanisms.

We observed reduced IL‐22 and phosphorylated STAT3 levels in the secretory endometrium of patients with PCOS and validated this finding in mice and organoids. Mechanistically, STAT3 directly binds to the *IGFBP5* promoter and activated its transcription, linking IL‐22 deficiency to impaired IGFBP5 expression. IGFBP5 supplementation rescued implantation failure by enhancing endometrial receptivity, whereas IGFBP5 depletion abolished the effects of IL‐22, establishing IGFBP5 as a key downstream effector of IL‐22‐STAT3 signaling. Together, our findings offer mechanistic insights into PCOS pathology, providing a new potential therapeutic target for improving implantation disorders.

IGFBP5 is highly expressed in the female reproductive system, but its biological function is relatively understudied. Previous studies have linked IGFBP5 to granulosa cell senescence and ovarian dysfunction [28, 29]. In addition, IGFBP5 suppresses trophoblast cell migration and invasion in the preeclamptic placenta, which may contribute to reduced litter size and increased morbidity in transgenic mice overexpressing *Igfbp5* [21, 30]. However, its role in endometrial function during embryo implantation is still poorly understood. Our results suggested that STAT3 binds to the *IGFBP5* promoter in the PCOS endometrium, directly controlling its expression. Furthermore, IL‐22‐mediated improvement of endometrial receptivity was disrupted when IGFBP5 was reduced, indicating that IL‐22 may exert its effect through the STAT3‐IGFBP5 pathway. IGFBP5 supplementation improved implantation defects in PCOS‐like mice by promoting the expression of endometrial receptivity‐related genes. These findings highlighted IGFBP5 as a critical determinant of endometrial receptivity and a potential protective factor in PCOS‐associated infertility.

As a member of the IGFBP family, IGFBP5 mainly regulates the binding of IGFs to IGF receptors, contributing to tumor progression [31, 32]. Although IGFBPs modulate IGF activity, IGFBP5 also influences disease through IGF‐independent mechanisms. For example, it promotes diabetic kidney disease by enhancing endothelial glycolysis [33] and, as a ligand for the receptor tyrosine kinase like orphan receptor 1 (ROR1), drives glioblastoma progression [34]. In this study, we found that IGFBP5 acts through an IGF‐independent mechanism in the endometrium. Specifically, IGFBP5 activated CREB phosphorylation, a transcription factor critical for embryo implantation and decidualization, and promoted *PGR* expression, thereby improving endometrial receptivity in PCOS‐like mice.

The secretory endometrium is primed for embryo implantation through the combined actions of progesterone and estrogen [35]. STAT3 has been implicated in implantation and proposed as a downstream mediator of progesterone receptor signaling [36]. Our results suggested that IL‐22‐STAT3 activation provides an additional cytokine‐driven mechanism that integrates immune and hormonal cues to regulate endometrial receptivity. Consistent with disrupted IL‐22‐STAT3 signaling in the PCOS secretory endometrium, PCOS‐derived endometrial organoids treated with estrogen and progesterone showed reduced pSTAT3 levels, indicating that altered epithelial STAT3 signaling may underline abnormal hormonal responses in PCOS endometrium. Notably, IL‐22 supplementation significantly activated STAT3 phosphorylation and increased uterine PR expression in both PCOS organoids and mouse models, suggesting that STAT3 regulates PR expression, thus affecting hormonal responses in the secretory endometrium. Interestingly, ChIP‐seq results of PR signaling suggested that STAT3 may also act as a downstream effector of PR [37, 38], and STAT3 and PR physically interact, as shown by co‐immunoprecipitation (Co‐IP) [39]. Therefore, defects in STAT3 signaling in response to altered PR levels may contribute to PCOS endometrial dysfunction. Whether STAT3 also functions as an upstream transcriptional regulator of PR within a feedback loop warrants further investigation.

In summary, this study revealed that reduced endometrial IL‐22 disrupted STAT3 signaling in the PCOS secretory endometrium and organoids. IL‐22 supplementation restored STAT3 phosphorylation, promoted its direct binding to the *IGFBP5* promoter, and reactivated IGFBP5 transcription, thereby alleviating endometrial dysfunction and implantation defects. Collectively, these findings highlighted the epithelial IL‐22‐STAT3‐IGFBP5 axis as a key determinant of endometrial receptivity. This mechanistic insight not only advances our understanding of PCOS pathophysiology but also highlights a potential endometrium‐focused therapeutic strategy for improving infertility in affected women.

## 4 Methods

### 4.1 Human Study Participants

Ethical approval for this study was granted by the Ethics Committee of Peking University Third Hospital granted ethical approval for the study (IRB00006761‐M2022696), and all participants provided written informed consent, in accordance with the Council for International Organizations of Medical Sciences. All volunteers recruited for this study are Han Chinese women. PCOS was based on the 2003 Rotterdam criteria, as described previously [18, 40]. The CON group included women with tubal occlusion or partners with azoospermia. Women with Cushing syndrome, thyroid disorders, 21‐hydroxylase deficiency, androgen‐secreting tumors, congenital adrenal hyperplasia, or hyperprolactinemia were excluded prior to PCOS diagnosis. Additional exclusion criteria included female genital malformations, intrauterine adhesions, submucosal uterine fibroids, history of genital tuberculosis, and untreated hydrosalpinx. All participants had not used oral contraceptives or other hormonal medications within three months before the study.

Endometrial tissues were collected at defined menstrual cycle phases based on the last menstrual period, serum LH peak, and transvaginal ultrasound‐confirmed ovulation. Proliferative‐phase endometrium was obtained on cycle days 5–14, and secretory‐phase endometrium was collected 5–8 days post‐ovulation. Tissue staging was confirmed by pathological examination.

### 4.2 Generation, Culture, and Passaging of Patient‐Derived Endometrial Organoids

As described previously [20], proliferative‐phase endometrium from the CON and PCOS groups (*n* = 10 per group) was collected on menstrual cycle days 5–14; their clinical characteristics are listed in Table 1. Endometrial tissues were enzymatically digested at 37°C using 1 mg/mL of collagenase IV (Sigma‐Aldrich, USA). After 30 min of digestion, the cell suspension was pipetted and sequentially filtered through 100 and 40 µm filters (Falcon, USA) to isolate the cell component. The resulting cells were centrifuged, resuspended in 30 µL ice‐cold Matrigel (Corning, USA), and plated into 48‐well plates. Expansion medium (ExM) was prepared as shown in Table S1 and changed every 3 d. For organoid passaging, organoids were placed on ice for 15 min and then pipetted up and down 300 times using an electronic pipettor to dissociate them. Organoids were imaged daily at passage 1, and their diameters were measured using ImageJ software. Hormone or IL‐22 treatments were performed at passage 2.

**TABLE 1:** Clinical characteristics of women in the Control and PCOS groups for endometrial organoid generation.

	Control (*n* = 10)	PCOS (*n* = 10)	*P* value
Age (year)	31.000 ± 4.243	30.900 ± 3.446	0.955
Body mass index (kg/m^2^)	21.688 ± 2.770	23.113 ± 3.571	0.332
Infertility time (year)	2.750 (1.250, 4.750)	5.000 (3.000, 5.750)	0.070
FSH (U/L)	7.203 ± 1.648	6.181 ± 1.947	0.221
LH (U/L)	6.500 (3.997, 7.268)	7.015 (4.655, 9.020)	0.705
Testosterone (µg/L)	0.690 (0.690, 0.883)	1.710 (0.880, 1.930)	0.132
Androstenedione (nmol/L)	6.575 (5.635, 7.740)	18.950 (10.250, 21.150)	0.016
Fasting glucose level (mmol/L)	5.130 ± 0.245	5.220 ± 0.601	0.666
Fasting insulin level (μIU/mL)	5.534 ± 1.703	10.359 ± 6.039	0.034
HOMA‐IR	1.261 ± 0.381	2.387 ± 1.418	0.026
AMH (µg/L)	3.445 (2.860, 4.542)	10.480 (7.595, 11.123)	0.001

FSH: Follicle stimulating hormone, LH: Luteinizing hormone, HOMA‐IR: Homeostatic Model Assessment of Insulin Resistance, AMH: Anti‐Mullerian hormone. Proliferative‐phase endometrium was collected obtained on menstrual cycle days 5–14. Tissue staging was confirmed by pathological examination.

### 4.3 Hormone Stimulation and IL‐22 Treatment of Endometrial Organoids

Supplementation of E2 (Sigma–Aldrich), MPA (Med Chem Express, USA), and cAMP (Selleck, USA) was performed as described previously [19]. For six days, endometrial organoids were either cultured in ExM or exposed to 10 nM E2. To replicate the sequential effects of estrogen and progesterone, organoids were primed with 10 nM E2 for two days, followed by treatment for four days with 10 nM E2 + 1 µM MPA + 1 µM cAMP (a component mediating progesterone‐dependent decidualization) [19, 41]. For IL‐22 supplementation, PCOS endometrial organoids were treated with 100 ng/mL IL‐22 (HY‐P7039, Lot: 336874, ED_50_ <1 ng/mL; Med Chem Express) for 48 h and collected for downstream applications.

### 4.4 Culture and Treatment of Cell Lines

HEK293T cells were obtained from the American Type Culture Collection (ATCC, CRL‐3216). Ishikawa cells (human endometrial epithelial cell line) were kindly provided by Procell (CL‐0283, Wuhan, China). JAr cells (trophoblastic tumor cells) were purchased from iCell Bioscience, Inc. (iCell‐h251, Shanghai, China). DMEM containing 10% fetal bovine serum (FBS; Gibco, USA) and 1% penicillin‐streptomycin (PS; Gibco, USA) was used to culture 293T cells. JAr cells were cultured in RPMI 1640 (Gibco, USA) supplemented with 10% FBS and 1% PS. Ishikawa cells were cultured in DMEM/F12 supplemented with 10% FBS and 1% PS. After treatment with DHT (GlpBio, USA) for 48 h, Ishikawa cells were collected for RNA extraction. Before co‐cultured with JAr spheroids, Ishikawa cells were primed under serum‐free conditions for 24 h with DHT (0.5 µM), DHT + IGFBP5 (100 ng/mL, HY‐P72378, Med Chem Express), DHT + IGF1 (50 ng/mL, HY‐P7018, Med Chem Express), DHT + IGFBP5 + IGF1, or DHT + IGFBP5 + NVP‐AEW541 (10 µM, HY‐50866, Med Chem Express) to eliminate interference from serum‐derived factors.

### 4.5 Co‐Culture of JAr Spheroids and Ishikawa Cells

To create JAr spheroids, 100 mm Petri dishes with their bottoms covered by 2% agarose gel were used to culture JAr cells. After 24–48 h, JAr spheroids with diameters of 100–200 µm were collected. Twenty JAr spheroids were transferred to each well containing Ishikawa cells primed with DHT, DHT + IGFBP5, DHT + IGF1, DHT + IGFBP5 + IGF1, or DHT + IGFBP5 + NVP‐AEW541, followed by 1 h of co‐culture. Non‐adherent spheroids were removed by gentle washing with DPBS for three times, and the adhesion rate was defined as the proportion of attached spheroids. Images of adherent JAr spheroids were captured using a fluorescence microscope.

### 4.6 Plasmid Transfection

STAT3‐overexpressing (STAT3‐OE) plasmids were obtained from Miaoling Bio (Wuhan, China) and confirmed by sequencing. Using Lipofectamine 3000 reagent (Thermo fisher, USA), 2 µg STAT3‐OE plasmids or empty plasmids (vector) were transfected into Ishikawa cells seeded in 6‐well plates. An untreated CON group was included to control for potential effects induced by the transfection procedure or reagents. Ishikawa cells were harvested for immunofluorescence staining and RNA and protein extraction.

### 4.7 Point Mutation and Expression of the IGFBP5 Promoter Plasmid

The proximal promoter sequence of the human *IGFBP5* gene was retrieved from the UCSC genome browser (https://genome.ucsc.edu/) and cloned into the pGL4.17 plasmid to construct a promoter reporter plasmid (IGFBP5‐WT). The binding sites of STAT3 in the *IGFBP5* gene promoter were predicted using the JASPAR database (https://jaspar.elixir.no), and the three predicted sites with the highest scores were selected for validation. The primer sequences for point mutations were designed using SnapGene software and are listed in Table S2. The *IGFBP5* promoter sequence with point mutations (IGFBP5‐Mut) was amplified by PCR using the promoter reporter plasmid as a template, yielding a product of 7,573 bp. The PCR products were identified by 1.0% agarose gel electrophoresis, followed by digestion with the *Dpn*I restriction enzyme (Thermo Fisher, USA) for 2 h. After digestion, the products were transformed into *Escherichia coli* DH5α competent cells and incubated overnight at 37°C. Single colonies were picked and inoculated into Lysogeny Broth supplemented with 50 µg/mL ampicillin, and shaken at 200 rpm overnight at 37°C. Subsequently, the bacterial cultures were collected for sequencing and plasmid extraction. The point‐mutated plasmids were extracted using a plasmid extraction kit (TIANGEN, Beijing, China).

### 4.8 Dual‐Luciferase Reporter Assay

Using Lipofectamine 3000 reagent, 293T cells were transfected with a Renilla luciferase reporter and either a WT or Mut IGFBP5 reporter construct, along with an empty vector or STAT3‐OE plasmids at a ratio of 1:1:100. After 48 h of transfection, luciferase activity in the cell lysates was measured using a dual‐luciferase reporter assay (Promega, USA) on a Centro LB960 luminometer (Berthold, Germany). Renilla luciferase activity was utilized as an internal reference to normalize the data.

### 4.9 Lentivirus Production

The *Igfbp5* gRNA (5’‐ TTTCGTTGAGGCAAACCCCG‐3’) was cloned into SF‐LV‐gRNA‐Cas9‐GFP vector. Lentivirus was packaged in HEK293T cells and concentrated by ultracentrifugation at 25000rpm for 2.5 h.

### 4.10 Animals and Study Design

The Animal Care and Use Committee of Peking University granted ethical approval for the study protocol (A2022146). Twenty‐one‐day‐old wild‐type C57BL/6J female mice were housed with unrestricted access to water and food in a specific pathogen‐free (SPF) facility under a regulated 12‐h light:12‐h dark cycle and controlled temperature. After two days of adaptation, pups from multiple independent litters were pooled and randomly distributed into the experimental groups to minimize litter effects.

To demonstrate the effect of IL‐22 on endometrial receptivity, three‐week‐old female C57BL/6J prepubertal mice were injected subcutaneously with DHEA (Sigma–Aldrich, 6 mg/100 g body weight; dissolved in sesame oil) daily for three weeks to establish the DHEA‐induced PCOS‐like mouse model, mice in the CON group received daily subcutaneous injections of sesame oil. For IL‐22 treatment, mice in the CON+IL‐22 and DHEA+IL‐22 group received intraperitoneal injections of IL‐22 (100 µg/kg) every day during the three weeks of DHEA treatment, while PBS was administered to CON and DHEA mice in parallel.

To explore the role of IGFBP5 in IL‐22‐mediated improvement of endometrial receptivity in PCOS‐like mice model, a lentivirus vector (vector) or lentivirus containing *Igfbp5* sgRNA (Sg‐*Igfbp5*) was injected into both uterine horns on day 1 of pregnancy (D1) in DHEA+IL‐22‐treated mice, the implantation and endometrial receptivity of which was compared with DHEA‐treated PCOS‐like mice.

To clarify the amelioration of IGFBP5 on impaired endometrial receptivity in PCOS, recombinant human IGFBP5 (2 mg/kg, HY‐P72378, Med Chem Express) was intraperitoneally injected to DHEA‐treated mice daily from D1 to day 4 of pregnancy (D4), with PBS injected as a control in the CON and DHEA group [18, 40]. The IGFBP5 doses were determined from studies published previously [42, 43].

Throughout the testing period for PCOS‐related phenotypes, the DHEA‐treated mice were given subcutaneous injections of DHEA every other day for an additional week to sustain the modeling effect. Endometrial function was measured as described previously [35]. After co‐caging, the presence of a vaginal semen plug indicated successful mating and was defined as the first day of pregnancy (D1). Mice were euthanized on D4 to collect uterine tissues for analysis of endometrial receptivity markers. In a separate cohort, on day 5 of pregnancy (D5), anesthetized mice were injected with 0.1 mL of 2% Chicago blue (Sigma, USA) via retro‐orbital injection to visualize and count ISs.

### 4.11 RNA Extraction and RNA‐Sequencing (RNA‐seq) Analysis

Human endometrial tissues, mouse uteri, endometrial organoids, and cells were collected for RNA extraction using TRIzol reagent (Life Technologies, USA). For RNA‐seq analysis, secretory‐phase endometrium samples were collected from the CON and PCOS groups, whose clinical baseline features are listed in Table 2 (*n* = 5 per group). Index‐coded libraries were prepared using a TruSeq PE Cluster Kit v3‐cBot‐HS (Illumina) and clustered on a cBot Cluster Generation System. Library sequencing was performed on an Illumina NovaSeq platform. In‐house Perl scripts were used to process raw FASTQ data for adapter trimming and quality filtering. The quality of the clean data was assessed using Q20, Q30, and GC content metrics, and high‐quality reads were retained for downstream analysis. Clean paired‐end reads were aligned to the human reference genome (hg38) using Hisat2 v2.0.5.

**TABLE 2:** Clinical characteristics of women in the Control and PCOS groups for RNA‐seq.

	Control (*n* = 5)	PCOS (*n* = 5)	*P* value
Age (year)	32.048 ± 2.783	31.545 ± 3.417	0.657
Body mass index (kg/m^2^)	21.648 ± 2.598	23.140 ± 2.652	0.136
Infertility time (year)	3.000 (2.000, 4.500)	3.000 (2.000, 4.000)	0.710
FSH (U/L)	6.544 ± 1.765	5.478 ± 0.873	0.070
LH (U/L)	4.000 (3.150, 4.980)	4.430 (3.815, 7.910)	0.155
Testosterone (µg/L)	0.690 (0.540, 0.700)	0.690 (0.690, 0.795)	0.324
Androstenedione (nmol/L)	4.730 (3.780, 6.930)	9.130 (7.110, 10.800)	0.007
Fasting glucose level ((mmol/L)	5.076 ± 0.570	5.107 ± 0.696	0.893
Fasting insulin level (μIU/mL)	3.868 ± 0.512	11.298 ± 5.870	0.047
HOMA‐IR	0.821 ± 0.137	2.655 ± 1.932	0.067
AMH (µg/L)	2.740 (1.925, 3.685)	6.710 (5.905, 13.275)	<0.001

FSH: Follicle stimulating hormone, LH: Luteinizing hormone, HOMA‐IR: Homeostatic Model Assessment of Insulin Resistance, AMH: Anti‐Mullerian hormone. Secretory‐phase endometrium was collected 5–8 days post‐ovulation. Tissue staging was confirmed by pathological examination.

To minimize inter‐individual variability in clinical samples and endometrial organoids used for transcriptomic sequencing, clinical samples were stringently matched for key clinical parameters, including age, body mass index (BMI), and infertility time (years). In addition, all endometrial organoid groups were subjected to identical treatment, culture conditions, and experimental procedures, thereby minimizing potential systematic bias. For downstream analyses, genes were first pre‐filtered to retain only those with a count of at least 10 in a minimal number of samples. Differential gene expression analysis was performed using DESeq2 in R, with DEGs defined as those with adjusted *p* < 0.05 and |log_2_FC| > 1. Enrichment analysis for upregulated and downregulated DEGs was performed separately using Metascape. The DEGs of the PCOS secretory endometrium and IL‐22‐treated PCOS endometrial organoids are displayed in the Data File.

### 4.12 cDNA Synthesis and Quantitative Real‐Time PCR Analysis

cDNA from human endometrial tissues, mouse uteri, and Ishikawa cells was synthesized with 1 µg of total RNA using the HiScript III All‐in‐one RT SuperMix (Vazyme, China). Real‐time qPCR analysis was performed using the primers listed in Table S3 and the Taq Pro Universal SYBR qPCR Master Mix (Vazyme). Relative gene expression levels were normalized to *GAPDH*/*Gapdh*.

### 4.13 Paraffin Embedding and Staining

Endometrial organoids were pre‐embedded in embedding reagent (20 mg/mL agarose in 60% glycerol and 40% distilled water). Human endometrium, mouse uterus, and endometrial organoids were fixed in 4% paraformaldehyde (PFA). After dehydration in a graded series of alcohol and xylene, tissues and organoids were infiltrated with melted paraffin twice for 2 h, then embedded in wax and sectioned at 5 µm thickness. For H&E staining, paraffin sections were dewaxed in xylene and rehydrated through a graded series of alcohol. Hematoxylin solution and 1% hydrochloric acid in absolute ethanol were used for nuclear staining and differentiation. After eosin staining for 15 s, sections were dehydrated and sealed. For periodic acid‐Schiff (PAS) staining, a PAS dye solution set (Servicebio, Wuhan, China) was applied to detect glycogen in paraffin sections. Images were acquired using a WS‐10 scanner (WISLEAP, China).

### 4.14 Immunofluorescence Staining

Endometrial organoids were collected and fixed in 4% PFA for 30 min at 30–35°C. STAT3‐OE plasmid‐transfected Ishikawa cells were fixed in 4% PFA prior to immunofluorescence staining. Paraffin‐embedded sections of human endometrium and mouse uterus were deparaffinized and rehydrated. For heat‐induced antigen retrieval, sections were immersed in 10 mM citric acid (pH 6.0) and microwaved at 1000 W for 8 min, and then at 300 W for 10 min. Triton X‐100 (0.1%, Sigma) in PBS was used for permeabilization. All samples, including organoids, cells, and paraffin sections, were blocked for 1 h with blocking buffer (2% BSA in PBS) and incubated with primary antibodies at 4°C for 12–16 h. Samples were washed and sequentially stained with the appropriate secondary antibodies, followed by staining with 1 mg/mL DAPI solution (Solarbio, China). Images were acquired using a Carl Zeiss LSM 880 confocal microscope. Information on the antibodies used is provided in Table S4. Quantification analysis was performed using the HALO 4.1 platform (Indica Labs).

### 4.15 Immunohistochemistry

Antigen retrieval was performed on paraffin sections as described above. Endogenous peroxidase was quenched with 30% hydrogen peroxide for 15 min. Sections were blocked and incubated with primary antibodies at 4°C for 12–16 h. After three washes, sections were incubated with the appropriate secondary antibodies for 1 h. Diaminobenzidine tetrahydrochloride solution (ZSGB‐Bio, China) was prepared according to the manufacturer's instructions and applied to tissue sections for 2–10 min, after which the slides were examined under a microscope. When optimal staining was achieved, sections were rinsed with water to stop the DAB reaction. Cell nuclei were counterstained with hematoxylin. Sections were then dehydrated, and mounted, and prepared for image acquisition. Each experiment included a corresponding negative control. For quantification, the HALO 4.1 platform (Indica Labs) was used to assess immunohistochemical staining in epithelial and stromal cells in human endometrium and mouse uterus. Positive cells were categorized by staining intensity, and a histochemical scoring (H‐score) was assigned. Data are presented as the percentage of positive cells or H‐scores.

### 4.16 Western Blot Analysis

Proteins were extracted from cells, endometrial organoids, human endometria, and mouse uteri using RIPA lysis buffer (Beyotime, China) supplemented with phenylmethylsulfonyl fluoride (PMSF, Med Chem Express) and a protease inhibitor cocktail (Cell Signaling Technology, USA). Protein samples were loaded onto 4–20% SDS‐PAGE gels (GenScript, Nanjing, China) and transferred to prepared PVDF membranes (Merck Millipore, Germany). Membranes were blocked with 5% nonfat milk prepared in TBST (Tris‐buffered saline supplemented with 0.1% Tween 20; Applygen, Beijing, China), then incubated with the appropriate primary and secondary antibodies for specific protein detection. Antibody information is summarized in Table S4. Western blot bands were detected and analyzed using ImageJ, with normalization to GAPDH or β‐ACTIN.

### 4.17 Detection of IL‐22 Levels

For protein extraction, secretory‐phase endometrium was collected from the CON (*n* = 21) and PCOS (*n* = 11) groups at 5–8 days post ovulation, confirmed by endometrial pathological examination. Baseline clinical features of the CON and PCOS groups are listed in Table 3. IL‐22 levels in the extracted endometrial protein samples were examined using human IL‐22 ELISA kits (ABclonal, China).

**TABLE 3:** Clinical characteristics of women in the Control and PCOS groups for measurement of endometrial IL‐22 protein level.

	Control (*n* = 21)	PCOS (*n* = 11)	*P* value
Age (year)	32.048 ± 2.783	31.545 ± 3.417	0.657
Body mass index (kg/m^2^)	21.648 ± 2.598	23.140 ± 2.652	0.136
Infertility time (year)	3.000 (2.000, 4.500)	3.000 (2.000, 4.000)	0.710
FSH (U/L)	6.544 ± 1.765	5.478 ± 0.873	0.070
LH (U/L)	4.000 (3.150, 4.980)	4.430 (3.815, 7.910)	0.155
Testosterone (µg/L)	0.690 (0.540, 0.700)	0.690 (0.690, 0.795)	0.324
Androstenedione (nmol/L)	4.730 (3.780, 6.930)	9.130 (7.110, 10.800)	0.007
Fasting glucose level (mmol/L)	5.076 ± 0.570	5.107 ± 0.696	0.893
Fasting insulin level (μIU/mL)	5.510 ± 1.808	10.900 ± 5.301	0.007
HOMA‐IR	1.246 ± 0.445	2.571 ± 1.567	0.019
AMH (µg/L)	2.740 (1.925, 3.685)	6.710 (5.905, 13.275)	<0.001

FSH: Follicle stimulating hormone, LH: Luteinizing hormone, HOMA‐IR: Homeostatic Model Assessment of Insulin Resistance, AMH: Anti‐Mullerian hormone. Secretory‐phase endometrium was collected 5–8 days post‐ovulation. Tissue staging was confirmed by pathological examination.

### 4.18 Correlation Analysis

Correlation between pSTAT3/STAT3 levels and IGFBP5 protein levels was performed using the Spearman's rank correlation test.

### 4.19 Statistical Analysis

GraphPad Prism version 8.0 was used to generate graphs for all experimental data. SPSS version 24 was used for all statistical analyses. For parametric data, results are presented as means ± SEM, and *P* value were determined using two‐tailed Student's *t*‐test or one‐way ANOVA followed by Tukey's post hoc test. For non‐parametric data, results are presented as medians with interquartile ranges, and *P* values were determined using the two‐tailed Mann–Whitney U‐test or Kruskal–Wallis test followed by Dunn's post hoc test.

## Author Contributions

Study design was performed by R. Li, Y. Pang, B. Liao, and C. Yun. Data acquisition, analysis, or interpretation was carried out by B. Liao, C. Yun, and H. Shan. The manuscript was drafted by B. Liao and C. Yun. Clinical samples were collected by B. Liao, W. Lian, and T. Peng, while clinical data were collected by H. Shan, M. Zhao, and K. Hu. Critical revision of the manuscript for important intellectual content was completed by B. Liao, C. Yun, H. Shan, Y. Wang, Y. Wang, and P. Zhou. Administrative, technical, or material support was provided by R. Li, Y. Pang, Y. Wang, P. Zhou, and X. Qin. Supervision was provided by R. Li, Y. Pang, Y. Wang, and P. Zhou. B. Liao, C. Yun, and H. Shan contributed equally to this work.

## Funding

This study is supported by the National Natural Science Foundation of China (82288102, 82301888, 82401917), National Key Research and Development Program of China (2025YFE0117400, 2025YFC2708000, 2024YFA1803000, 2022YFC2702500), Scientific Research Innovation Capability Support Project for Young Faculty (ZYGXQNJSKYCXNLZCXM‐H2), The Xinjiang Production and Construction Corps Guiding Science and Technology Program (2023ZD008) and Excellent Doctoral Innovation Fund of Peking University Health Science Center (BMU2025BSS0011).

## Conflicts of Interest

The authors declare no conflicts of interest.

## Supporting information




**Supporting File 1**: advs74683‐sup‐0001‐SuppMat.docx.

**Supporting File 2**: advs74683‐sup‐0002‐Data.zip.

**Supporting File 3**: advs74683‐sup‐0003‐Supplemental figure‐Original images for Western Blot.pdf.

## Data Availability

The data and materials supporting the conclusions of this article are available from the corresponding authors on request.
