# Supplementary material for: IGFBP5 Restores Endometrial Receptivity and Rescues Implantation Failure in Polycystic Ovary Syndrome
Source: Adv Sci (Weinh). 2026 Mar 12;13(27):e20455. doi: 10.1002/advs.202520455 (PMC13170201; doi:10.1002/advs.202520455)
Supplement: Supplementary file 1 — Supporting File 1: advs74683‐sup‐0001‐SuppMat.docx. [file ADVS-13-e20455-s001.docx]

Supporting Information

IGFBP5 Restores Endometrial Receptivity and Rescues Implantation Failure in Polycystic Ovary Syndrome

*Baoying Liao, Chuyu Yun, Hongying Shan, Yuqian Wang, Xiunan Chen, Weisi Lian, Tianliu Peng, Min Zhao, Xunsi Qin, Kailun Hu, Ping Zhou*, Yue Wang*, Yanli Pang*, Rong Li**

**Supplementary figures**


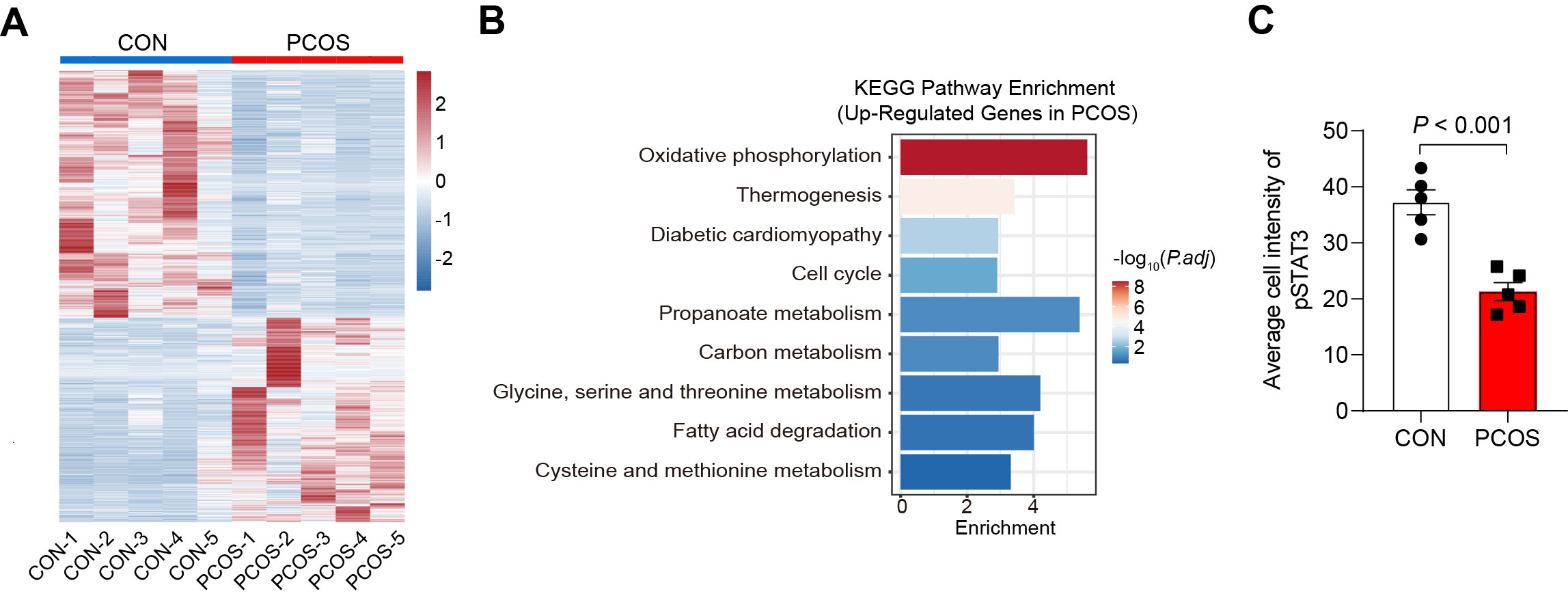


**Figure S1. Transcriptional signature of PCOS secretory endometrium.** (A) Heatmap of DEGs (|log2FC| > 1, adj. *p* < 0.05) between CON and PCOS secretory endometrium. (B) Pathways significantly enriched by KEGG analysis of upregulated DEGs in PCOS secretory endometrium compared with the CON group. (C) Quantitative results of pSTAT3 protein in CON and PCOS secretory endometrium, *n* = 5 per group. For C, data are presented as mean ± SEM, the *P* value was determined by a two-tailed Student’s *t*-test.


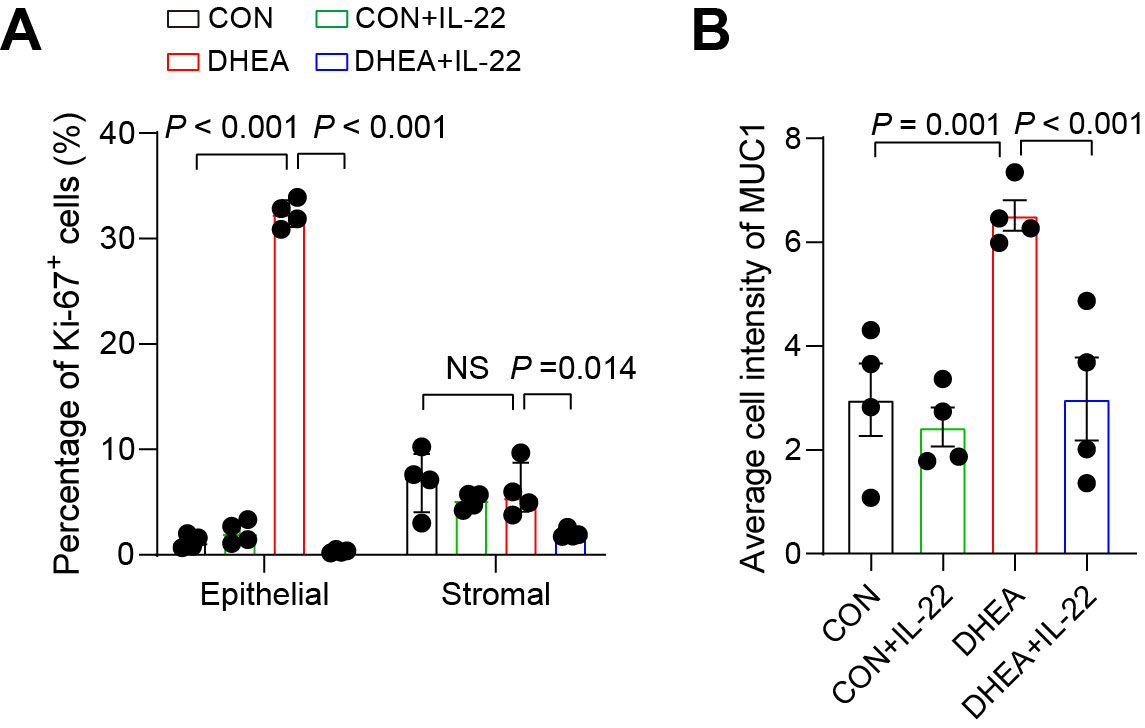


**Figure S2. IL-22 alleviates endometrial dysfunction in PCOS-like mice.** (A) The percentage of Ki67^+^ cells in endometrial epithelial and stromal cells in the mice uterus on day 4, *n =* 4 per group. (B) Quantitative results of MUC1 protein in mice uterus on day 4 of pregnancy, *n =* 4 per group. For A and B, data are presented as mean ± SEM, the *P* value was determined by one-way ANOVA with Tukey’s multiple comparison post hoc test.


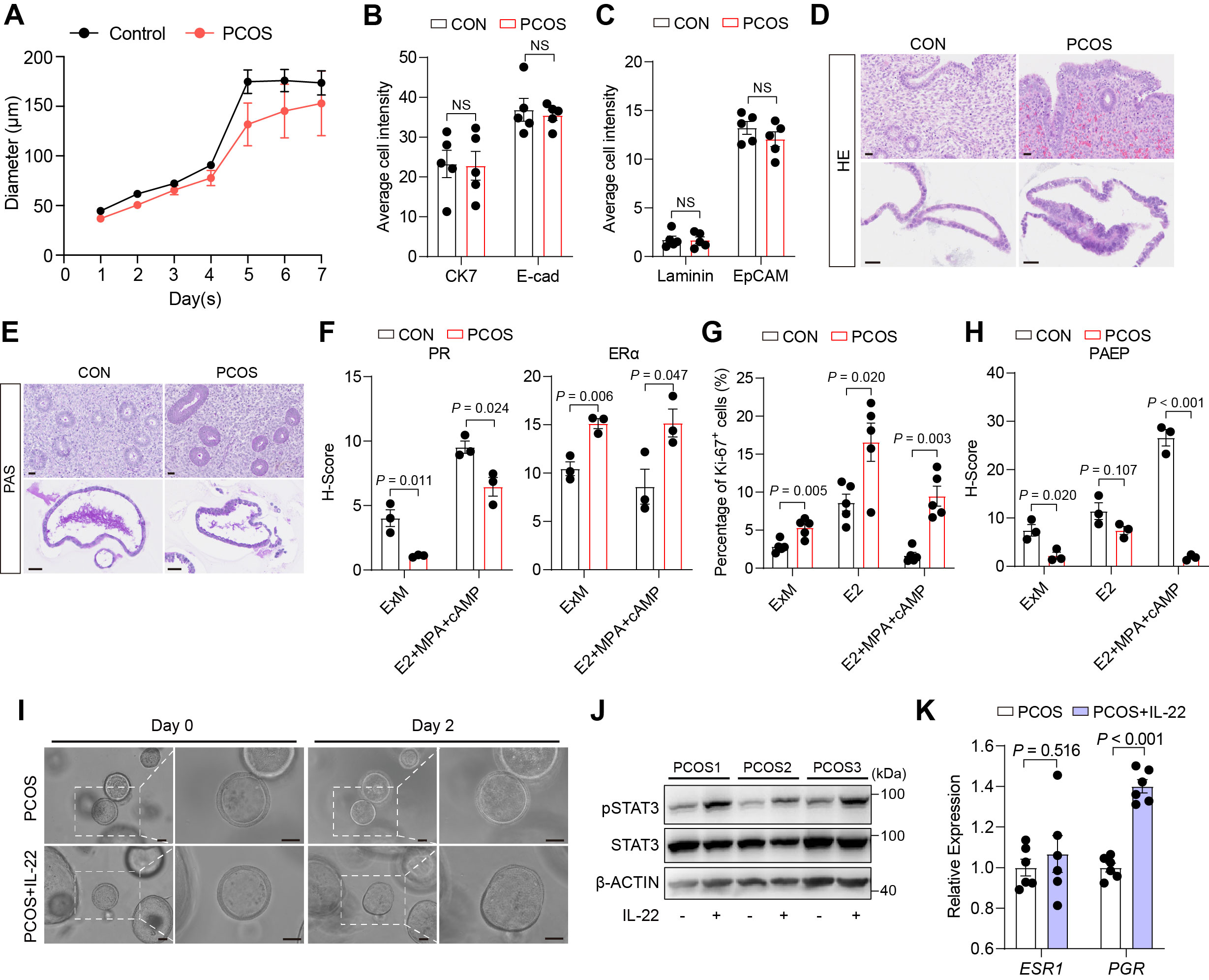


**Figure S3. Endometrial organoids recapitulate the transcriptional signature of the endometrium *in vivo*.** (A) Diameter statistics, *n =* 10 per group. (B) Quantitative results of cytokeratin-7 (CK7), E-cadherin (E-cad) protein in endometrial organoids, *n* = 5 per group. (C) Quantitative analysis result of cell basement membrane marker Laminin and EpCAM in endometrial organoids, *n* = 5 per group. (D) Hematoxylin-eosin (HE) result of endometrial tissue and organoids in the CON and PCOS groups, scale bars: 50 μm. (E) Periodic acid Schiff (PAS) staining of endometrial tissue and organoids in the CON and PCOS groups, scale bars: 50μm. (F) Histochemical scoring assessment (H-Score) of ERα and PR in hormone-treated endometrial organoids, *n* = 3 per group. (G) The percentage of Ki67^+^ cells in hormone-treated endometrial organoids, *n =* 5 per group. (H) Histochemical scoring assessment (H-Score) of PAEP in hormone-treated endometrial organoids, *n* = 3 per group. (I-J) Representative images (I) and representative immunoblot of pSTAT3 and STAT3 levels (J) for endometrial organoids in the PCOS and PCOS+IL-22 groups, *n =* 3 per group, scale bars: 50 μm; (K) Relative mRNA levels of *ESR1* and *PGR* in PCOS and PCOS+IL-22 endometrial organoids, *n =* 6 per group. For B, C, F-H and K, data are presented as mean ± SEM, the *P* value was determined by a two-tailed Student’s *t*-test.


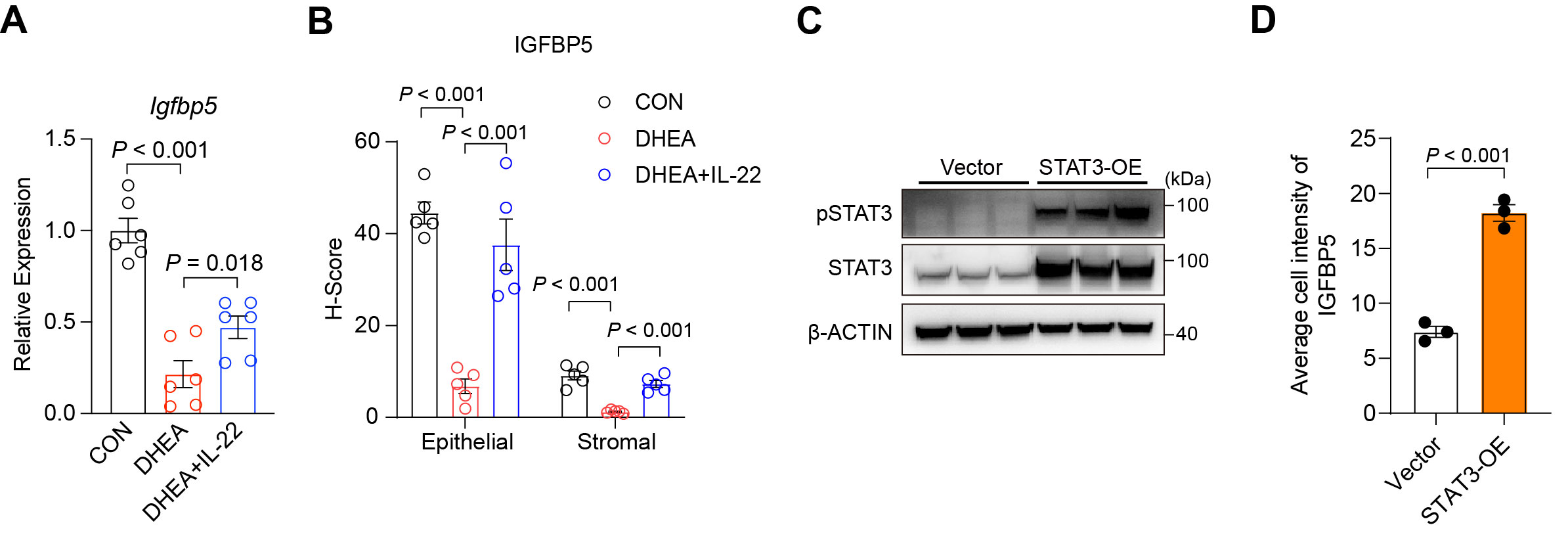


**Figure S4. IL-22-STAT3 activated *Igfbp5* gene transcription.** (A) *Igfbp5* mRNA levels in CON, DHEA and DHEA + IL-22 mice uterus on day 4 of pregnancy, *n =* 6 per group. (B) Histochemical scoring assessment (H-Score) of IGFBP5 protein in mice uterus on day 4 of pregnancy, *n =* 5 per group. (C) Representative immunoblots of pSTAT3 and STAT3 in STAT3-OE Ishikawa cells. (D) Quantitative results of IGFBP5 protein in STAT3-OE Ishikawa cells, *n =* 3 per group. Data are presented as mean ± SEM. For A and B, the *P* value was determined by one-way ANOVA with Tukey’s multiple comparison post hoc test. For D, the *P* value was determined by a two-tailed Student’s *t*-test.


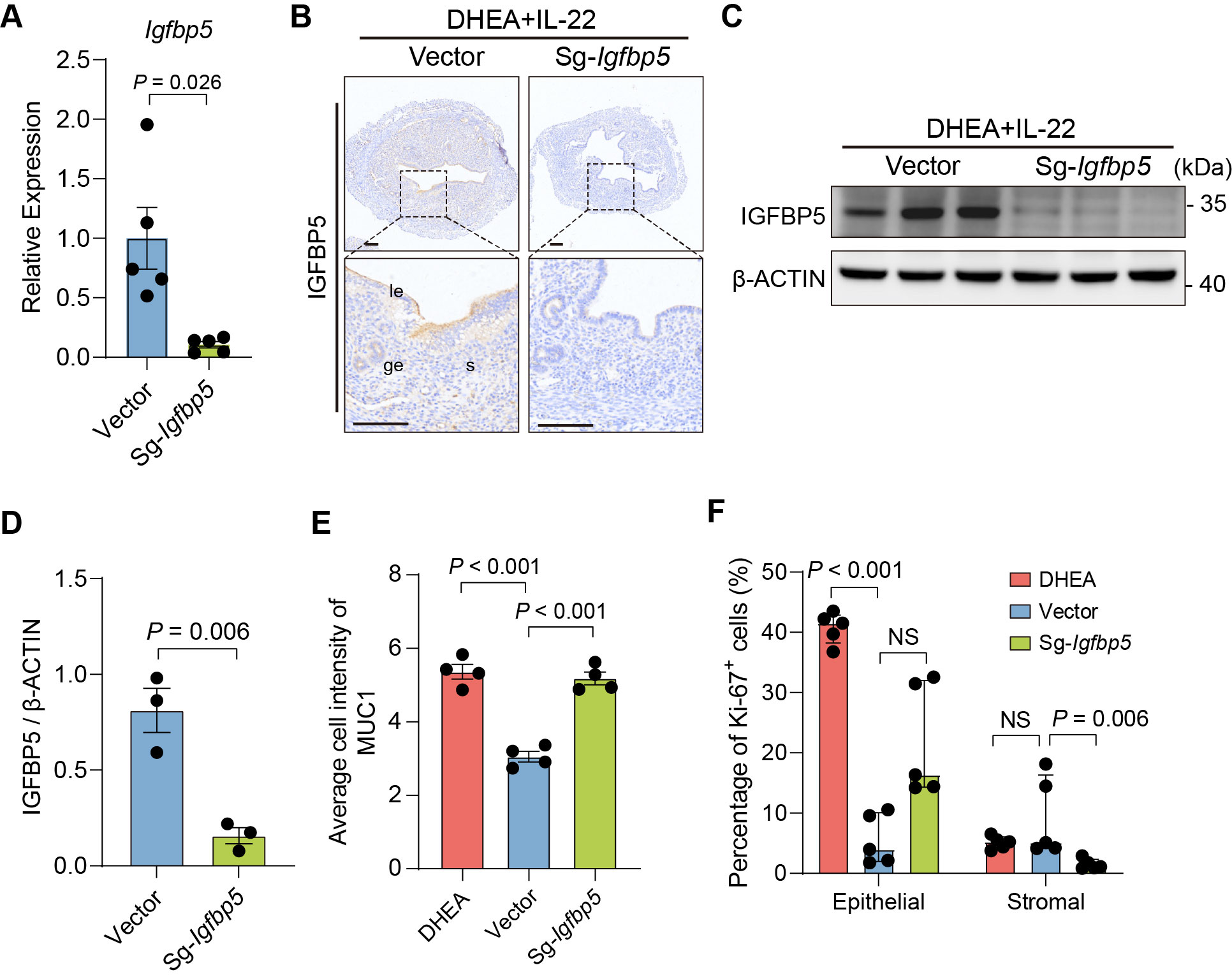


**Figure S5. IGFBP5 expression was down-regulated by lentivirus injection.** (A) *Igfbp5* mRNA levels in DHEA + IL-22 mice uterus with and without lentivirus injection on day 4 of pregnancy, *n =* 5 per group; (B) Representative immunochemistry staining image of IGFBP5 in DHEA + IL-22 mice uterus with and without lentivirus injection on day 4 of pregnancy, scales: 100 μm. le, luminal epithelium; ge, glandular epithelium; s, stroma. (C-D) Representative immunoblots (C) and quantitative analysis (D) of IGFBP5 in DHEA + IL-22 mice uterus with and without lentivirus injection on day 4 of pregnancy, *n =* 3 per group. (E) Quantitative results of MUC1 protein in mice uterus on day 4 of pregnancy, *n =* 4 per group. (F) The percentage of Ki67^+^ cells in endometrial epithelial and stromal cells in the murine uterus on day 4, *n =* 5 per group. For A and D, data are presented as mean ± SEM, the *P* value was determined by a two-tailed Student’s *t*-test. For E, data are presented as mean ± SEM, the *P* value was determined by one-way ANOVA with Tukey’s multiple comparison post hoc test. For F, data are presented as medians with interquartile ranges, the *P* value was determined Kruskal–Wallis test followed by Dunn’s post hoc test.


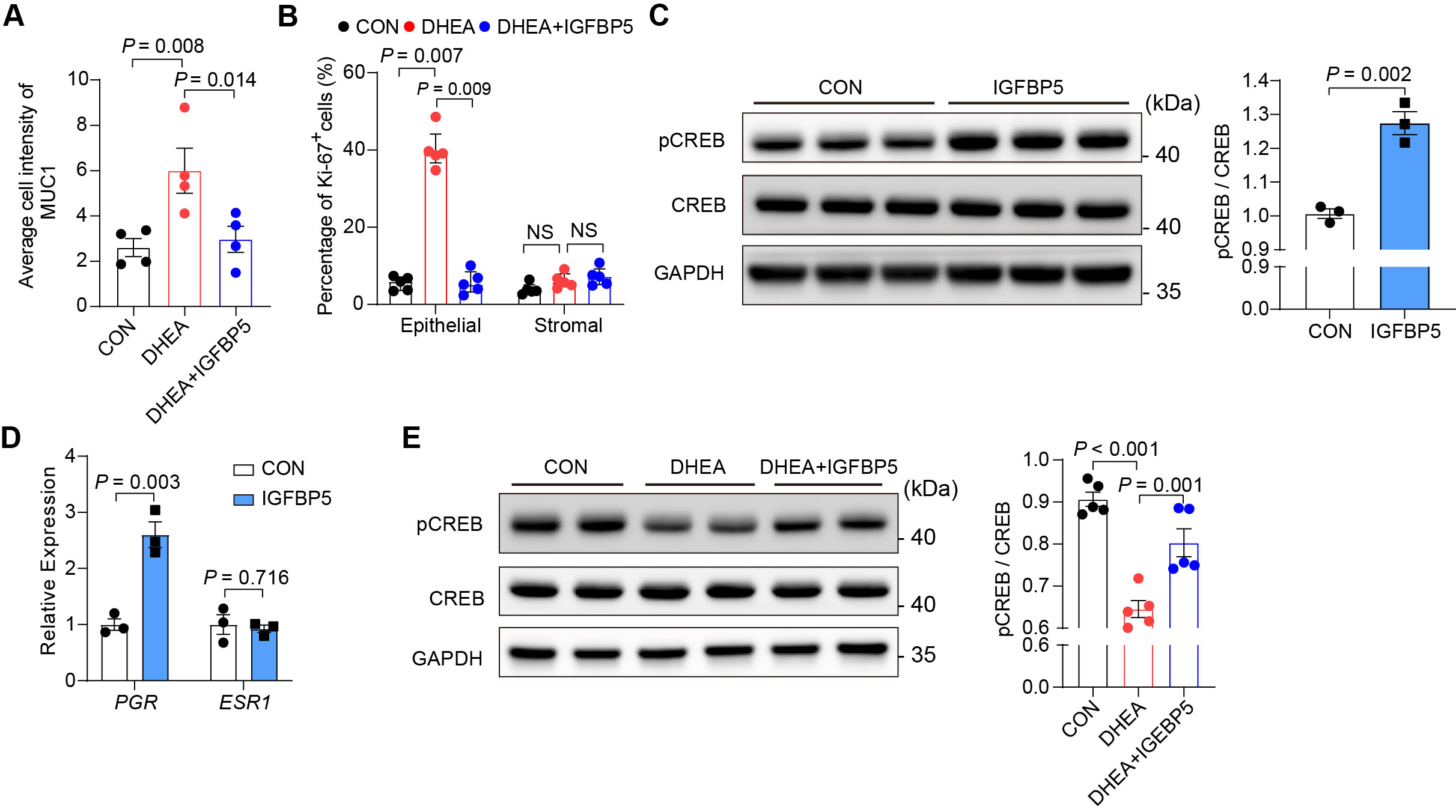


**Figure S6. IGFBP5 promotes *PGR* expression by activating CREB.** (A) Quantitative results of MUC1 protein in mice uterus on day 4 of pregnancy, *n =* 4 per group. (B) The percentage of Ki67^+^ cells in endometrial epithelial and stromal cells in the murine uterus on day 4, *n =* 5 per group. (C) Representative immunoblots and quantitative analysis of pCREB and CREB in Ishikawa cells treated with or without IGFBP5 (100 ng/ml), *n* = 3 per group. (D) Relative expression of *PGR* and *ESR1* in Ishikawa cells treated with or without IGFBP5 (100 ng/ml), *n* = 3 per group. (E) Representative immunoblots and quantitative analysis of pCREB and CREB in in CON, DHEA and DHEA + IGFBP5 mice uterus on day 4 of pregnancy, *n* = 5 per group. For A, B and E, data are presented as mean ± SEM, the *P* value was determined by one-way ANOVA with Tukey’s multiple comparison post hoc test. For C and D, data are presented as mean ± SEM, the *P* value was determined by a two-tailed Student’s *t*-test.

**Supplementary Tables**

**Table S1**. Composition of Expansion medium (ExM) for organoid.

| Component | Concentration | Catalog number | Source |
| --- | --- | --- | --- |
| N2 | 1% | 17502048 | ThermoFisher, USA |
| B27 | 2% | 17504044 | ThermoFisher, USA |
| Glutamax | 1% | 35050061 | Gibco, USA |
| ITS | 1% | 41400045 | ThermoFisher, USA |
| PS | 1% | 15140122 | Gibco, USA |
| N-Acetylcysteine | 1.25mM | A0737 | Sigma-Aldrich, USA |
| Nicotinamide | 10 nM | N0636 | Sigma-Aldrich, USA |
| Y-27632 | 10 μM | HY-10583 | Med Chem Express, USA |
| A83-01 | 0.5 μM | HY-10432 | Med Chem Express, USA |
| EGF | 50 ng/ml | C029 | Novoprotein, China |
| HGF | 50 ng/ml | CJ72 | Novoprotein, China |
| FGF10 | 100 ng/ml | CR11 | Novoprotein, China |
| Noggin | 100 ng/ml | CB89 | Novoprotein, China |
| R-Spondin 1 | 100 ng/ml | CX83 | Novoprotein, China |
| DMEM/F12 |  | 12634028 | Gibco, USA |

**Table S2**. Primer sequences used for point mutation.

| *IGFBP5*-MUT1 |
| --- |
| Forward 5’- CGGTTCCTGGTCAGGACCTCGGAACCTACGGTCCTGTGGCCCTGTCAGC -3’ |
| Reverse 5’- GCTGACAGGGCCACAGGACCGTAGGTTCCGAGGTCCTGACCAGGAACCG-3’ |
| *IGFBP5*-MUT2 |
| Forward5’-GAACTGAACGAGCAAGAAAAAATATTGGAGGTCCCGCAGTCCTCCAGAAAGAATTC-3’ |
| Reverse 5’-GAATTCTTTCTGGAGGACTGCGGGACCTCCAATATTTTTTCTTGCTCGTTCAGTTC-3’ |
| *IGFBP5*-MUT3 |
| Forward 5’- GCATCCTTGCCTGAGTTGGGGTGGTTTCCGCTCAAATTGCAGCTACAAAC-3’ |
| Reverse 5’- GTTTGTAGCTGCAATTTGAGCGGAAACCACCCCAACTCAGGCAAGGATGC-3’ |

**Table S3**. Primer sequences used in quantitative real-time PCR analysis.

| Target genes | Primer sequence |
| --- | --- |
| *Gapdh* (mouse) | Forward 5’- AGGTCGGTGTGAACGGATTTG -3’ |
|  | Reverse 5’- TGTAGACCATGTAGTTGAGGTCA -3’ |
| *Esr1* (mouse) | Forward 5’- TCTGCCAAGGAGACTCGCTACT -3’ |
|  | Reverse 5’- GGTGCATTGGTTTGTAGCTGGAC -3’ |
| *Pgr* (mouse) | Forward 5’- CTACTCGCTGTGCCTTACCATG-3’ |
|  | Reverse 5’- CTGGCTTTGACTCCTCAGTCCT-3’ |
| *Lif* (mouse) | Forward 5’- AGCTATGTGCGCCTAACATGA-3’ |
|  | Reverse 5’- CGACCATCCGATACAGCTCC-3’ |
| *Muc1* (mouse) | Forward 5’- AGCCCCTATGAGGAGGTTTCG -3’ |
|  | Reverse 5’- AAGTGGTCACCACAGCTGGG -3’ |
| *Hand2* (mouse) | Forward 5’- GCAGGACTCAGAGCATCAACA-3’ |
|  | Reverse 5’- AGGTAGGCGATGTATCTGGTG-3’ |
| *Hoxa10* (mouse) | Forward 5’- GGCAGTTCCAAAGGCGAAAAT-3’ |
|  | Reverse 5’- GTCTGGTGCTTCGTGTAAGGG-3’ |
| *Areg* (mouse) | Forward 5’- GGGGACTACGACTACTCAGAG-3’ |
|  | Reverse 5’- TCTTGGGCTTAATCACCTGTTC-3’ |
| *Ihh* (mouse) | Forward 5’- CGGCTTCGACTGGGTGTATTAC-3’ |
|  | Reverse 5’- AGGAAAGCAGCCACCTGTCTTG-3’ |
| *Igfbp5* (mouse) | Forward 5’-CCCTGCGACGAGAAAGCTC -3’ |
|  | Reverse 5’-GCTCTTTTCGTTGAGGCAAACC -3’ |
| *GAPDH* (human) | forward 5′- GGAGCGAGATCCCTCCAAAAT-3′ |
|  | reverse 5′-GGCTGTTGTCATACTTCTCATGG -3′ |
| *PAEP* (human) | Forward 5’- GAGATCGTTCTGCACAGATGG-3’ |
|  | Reverse 5’- CGTTCGCCACCGTATAGTTGAT-3’ |
| *SPP1* (human) | Forward 5’- GAAGTTTCGCAGACCTGACAT-3’ |
|  | Reverse 5’- GTATGCACCATTCAACTCCTCG-3’ |
| *IHH* (human) | Forward 5’- AGACCGCGACCGCAATAAG-3’ |
|  | Reverse 5’- GCCTTTGACTCGTAATACACCCA-3’ |
| *LIF* (human) | Forward 5’- CCAACGTGACGGACTTCCC-3’ |
|  | Reverse 5’- TACACGACTATGCGGTACAGC-3’ |
| *HSD17B2* (human) | Forward 5’- ATGGAAAGGCTGGCATCTTATG-3’ |
|  | Reverse 5’- CCTCCAGGTTGGATGGAAGC-3’ |
| *IGFBP5* (human) | Forward 5’- TGACCGCAAAGGATTCTACAAG-3’ |
|  | Reverse 5’- CGTCAACGTACTCCATGCCT-3’ |
| *ESR1* (human) | Forward 5’- GCTTACTGACCAACCTGGCAGA-3’ |
|  | Reverse 5’- GGATCTCTAGCCAGGCACATTC-3’ |
| *PGR* (human) | Forward 5’- ACCCGCCCTATCTCAACTACC-3’ |
|  | Reverse 5’- AGGACACCATAATGACAGCCT-3’ |

**Table S4.** Antibody information.

| Antibody | Catalog number | Dilution | Source |
| --- | --- | --- | --- |
| **Immunofluorescence** |  |  |  |
| Laminin | ab11575 | 1:200 | Abcam, Cambridge, UK |
| EpCAM | ab187372 | 1:200 | Abcam, Cambridge, UK |
| E-cadherin | ab40772 | 1:200 | Abcam, Cambridge, UK |
| Cytokeratin-7 | ab181591 | 1:200 | Abcam, Cambridge, UK |
| Ki-67 | ab15580 | 1:200 | Abcam, Cambridge, UK |
| MUC1 | ab109185 | 1:200 | Abcam, Cambridge, UK |
| IGFBP5 | mab8751 | 1:100 | R&D system, Minnesota, USA |
| **Immunohistochemistry** |  |  |  |
| ERα | ab108398 | 1:200 | Abcam, Cambridge, UK |
| PR | 8757 | 1:200 | Cell Signaling Technology, USA |
| PAEP | ab270454 | 1:200 | Abcam, Cambridge, UK |
| **Western Blotting** |  |  |  |
| COX2 | ab179800 | 1:1000 | Abcam, Cambridge, UK |
| pSTAT3 | 9145 | 1:1000 | Cell Signaling Technology, USA |
| STAT3 | 9139 | 1:1000 | Cell Signaling Technology, USA |
| pCREB | 9198 | 1:1000 | Cell Signaling Technology, USA |
| CREB | 9197 | 1:1000 | Cell Signaling Technology, USA |
| GAPDH | ab9485 | 1:2000 | Abcam, Cambridge, UK |
| β-ACTIN | ab6276 | 1:2000 | Abcam, Cambridge, UK |
| **Secondary antibody** |  |  |  |
| HRP-labeled Goat Anti-Rabbit IgG(H+L) | A0208 | 1:2000 | Beyotime, Shanghai, China |
| HRP-labeled Goat Anti-Mouse IgG(H+L) | A0216 | 1:2000 | Beyotime, Shanghai, China |
| Goat anti-Mouse IgG (H+L), Alexa Fluor™ 488 | A-10680 | 1:200 | Thermo Fisher Scientific, USA |
| Goat anti-Rabbit IgG (H+L), Alexa Fluor™ 555 | A-21428 | 1:200 | Thermo Fisher Scientific, USA |
