# Supplementary figures and images for: IGFBP5 Restores Endometrial Receptivity and Rescues Implantation Failure in Polycystic Ovary Syndrome
Source: Adv Sci (Weinh). 2026 Mar 12;13(27):e20455. doi: 10.1002/advs.202520455 (PMC13170201; doi:10.1002/advs.202520455)

Figure 3I

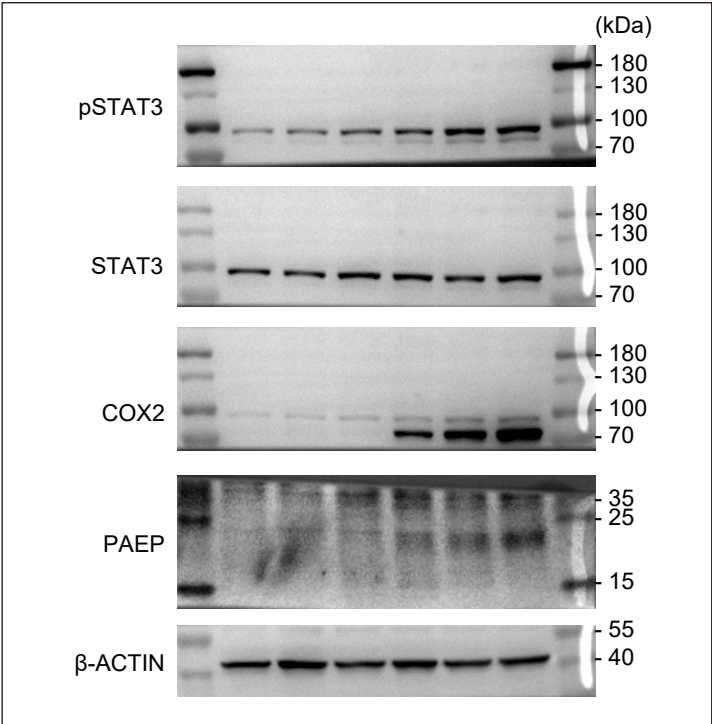

Figure 4C

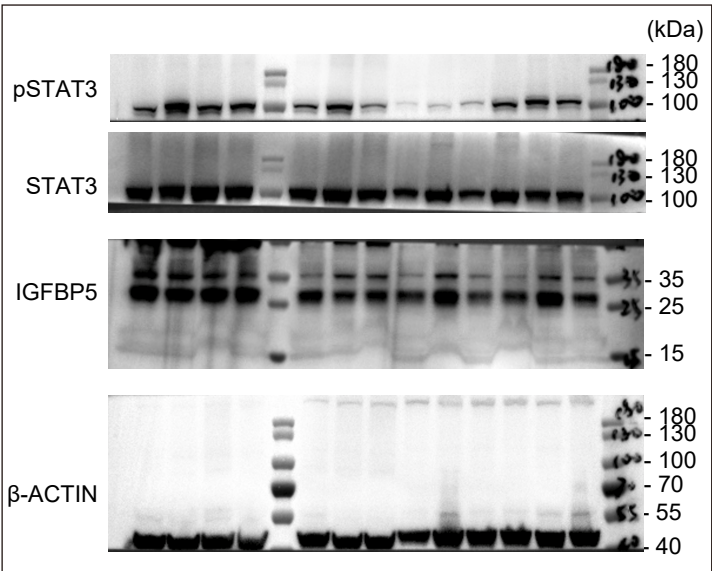

Figure S3J

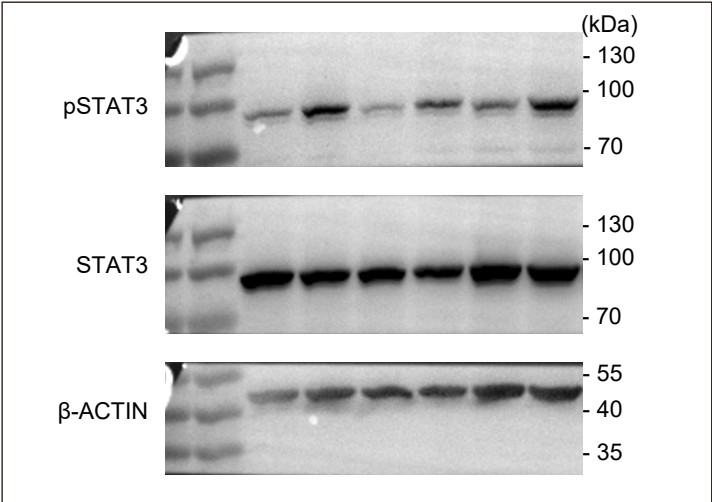

Figure S4C

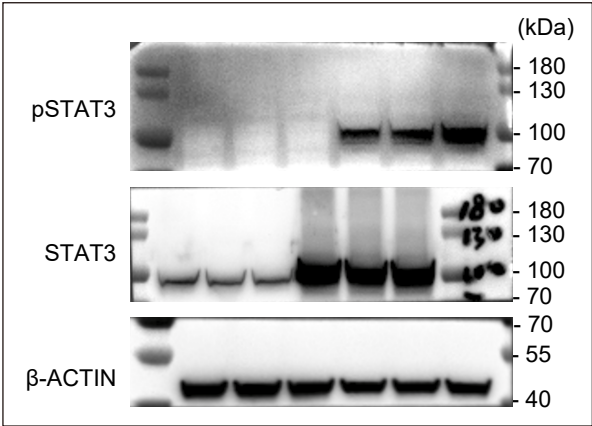

Figure S5C

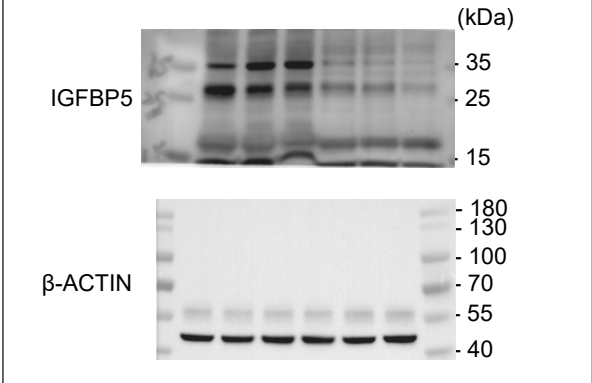

Figure S6C

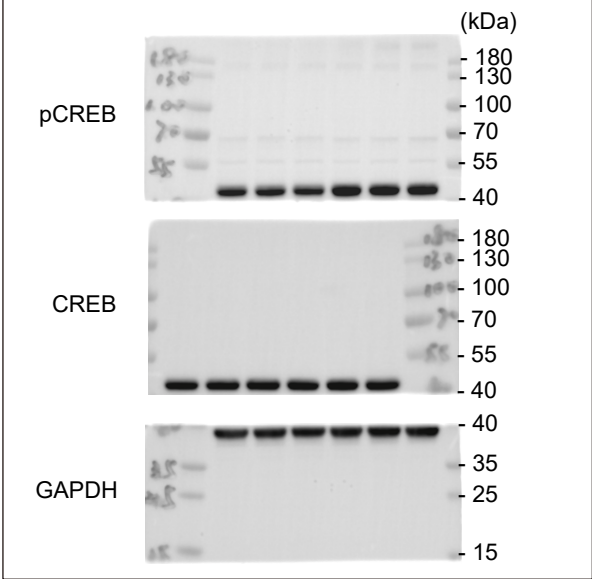

Figure S6E

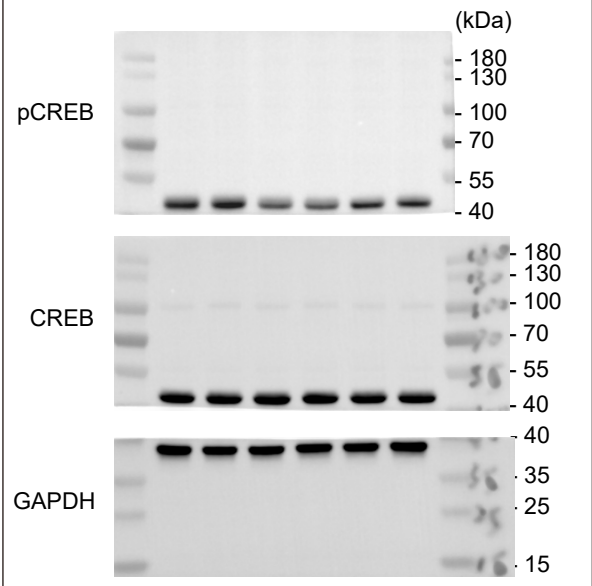

Supplement: Supplementary file 3 — Supporting File 3: advs74683‐sup‐0003‐Supplemental figure‐Original images for Western Blot.pdf. [file ADVS-13-e20455-s003.pdf]
